# Visualizing Pyrazinamide Action by Live Single-Cell Imaging of Phagosome Acidification and Mycobacterium tuberculosis pH Homeostasis

**DOI:** 10.1128/mbio.00117-22

**Published:** 2022-03-24

**Authors:** Pierre Santucci, Beren Aylan, Laure Botella, Elliott M. Bernard, Claudio Bussi, Enrica Pellegrino, Natalia Athanasiadi, Maximiliano G. Gutierrez

**Affiliations:** a Host-Pathogen Interactions in Tuberculosis Laboratory, The Francis Crick Institute, London, United Kingdom; Weill Cornell Medical College

**Keywords:** tuberculosis, microenvironments, antibiotics, intracellular pharmacokinetics, human macrophages, *Mycobacterium tuberculosis*

## Abstract

Mycobacterium tuberculosis segregates within multiple subcellular niches with different biochemical and biophysical properties that, upon treatment, may impact antibiotic distribution, accumulation, and efficacy. However, it remains unclear whether fluctuating intracellular microenvironments alter mycobacterial homeostasis and contribute to antibiotic enrichment and efficacy. Here, we describe a live dual-imaging approach to monitor host subcellular acidification and M. tuberculosis intrabacterial pH. By combining this approach with pharmacological and genetic perturbations, we show that M. tuberculosis can maintain its intracellular pH independently of the surrounding pH in human macrophages. Importantly, unlike bedaquiline (BDQ), isoniazid (INH), or rifampicin (RIF), the drug pyrazinamide (PZA) displays antibacterial efficacy by disrupting M. tuberculosis intrabacterial pH homeostasis *in cellulo*. By using M. tuberculosis mutants, we confirmed that intracellular acidification is a prerequisite for PZA efficacy *in cellulo*. We anticipate this imaging approach will be useful to identify host cellular environments that affect antibiotic efficacy against intracellular pathogens.

## INTRODUCTION

Tuberculosis (TB), caused by Mycobacterium tuberculosis, remains one of the deadliest infectious diseases worldwide (1). In 2020, it was estimated that almost 10 million people developed the active form of the disease and 1.3 million people died from TB, and recent data indicate that the COVID-19 pandemic is disrupting access to TB care and treatment (1).

Drug-susceptible TB treatment relies on a standard chemotherapy regimen that includes four first-line antibiotics, rifampicin (RIF), isoniazid (INH), ethambutol (EMB), and pyrazinamide (PZA), which are administered over a period of at least 6 months (1). This extensive treatment is often associated with side effects and toxicity, affecting compliance and contributing to the emergence of antibiotic-resistant strains (2). In that context, it is crucial to better understand how current anti-TB chemotherapies work to develop a new generation of efficient, fast-acting, and compliance-friendly treatments.

TB is a complex disease in which M. tuberculosis infection is mainly characterized by the formation of heterogeneous pulmonary granulomatous lesions that evolve dynamically over time (3, 4). Inside these highly structured cellular aggregates, which may also contain necrotic material, alveolar macrophages constitute the primary niche used by the tubercle bacilli to replicate (5). To survive and replicate within host macrophages, M. tuberculosis has successfully developed multiple strategies to counteract host cell defense mechanisms (6). Among them, both modulation and subversion of phagosome maturation and its ability to survive within acidic and hydrolytic microenvironments have been demonstrated to be crucial for the intracellular lifestyle of M. tuberculosis (7–12). In addition, M. tuberculosis can damage the phagosome through the action of its type VII secretion system ESX-1 and cell wall-associated phthiocerol dimycocerosate (PDIM) lipids to access the pH-neutral, nutrient-rich cytosol (13–19).

Because M. tuberculosis is localized in several intracellular niches, the generation of new drug regimens should consider the efficient targeting of M. tuberculosis intracellular populations residing within host cells. Ideally, anti-TB chemotherapy must include antibiotics with pharmacokinetic properties that allow agents to (i) penetrate lung tissue and infected cells, (ii) reach the wide-ranging subcellular compartments in which M. tuberculosis resides, and (iii) be active in these specific microenvironments to finally display optimal antibacterial efficacy. Understanding how subcellular environments affect bacterial fitness (20, 21) but more importantly antibiotic localization, exposure, and consequently efficacy against intracellular pathogens is crucial, and these areas have only recently begun to be investigated (22, 23).

Cell compartment-specific consideration of bioactivity is of particular importance for antibiotics such as the front-line drug PZA, which was demonstrated to be highly potent against the tubercle bacilli within infected mice but did not display activity under standard culture conditions *in vitro* (24–27). Indeed, *in vitro* PZA requires an acidic pH below 5.5 to be effective against M. tuberculosis (25, 28, 29). In this widely accepted pH-dependent mechanism of action, PZA enters the bacteria by diffusion and is converted by the PncA enzyme to form the deprotonated negatively charged total pyrazinoic acid [POA^(−)^] anion. This POA^(−)^ anion is then actively exported into the extracellular milieu. If M. tuberculosis faces an acidic environment, POA^(−)^ acquires a hydrogen cation to form the neutral protonated pyrazinoic acid (HPOA) molecule. This protonated form is able to diffuse across the bacterial envelope to finally disrupt intrabacterial pH homeostasis and membrane potential (30). In that context, several independent studies proposed that POA molecules accumulate dependently on the external pH *in vitro* and further collapse M. tuberculosis intrabacterial pH homeostasis (29, 31–33). However, this proposed pH-dependent mode of action of PZA/POA, where molecules acidify the M. tuberculosis cytoplasm, has been challenged (34, 35). Alternative pH-independent mechanisms underlying PZA/POA efficacy have been proposed (36, 37) whereby POA^(−)^ acting at neutral pH can competitively inhibit the aspartate decarboxylase PanD and enhance its targeted degradation via the Clp system, thus impairing the biosynthesis of coenzyme A (38–41). In this model, PZA/POA molecules are active regardless of the environmental pH surrounding M. tuberculosis and do not act as intrabacterial pH-collapsing agents. Overall, it is notable that despite PZA being used as a front-line drug for almost 70 years in the clinic, the molecular and cellular mechanisms underlying PZA efficacy remain unclear (37).

By using correlative light electron ion microscopy (CLEIM) approaches, we showed that PZA/POA molecules require phagosomal residency and acidification to efficiently accumulate within M. tuberculosis and display optimal activity within human macrophages (23). M. tuberculosis can transit through neutral and acidic environments multiple times during the infection cycle (18, 42); however, it remains unclear how these spatial and temporal changes affect the intracellular activity of PZA and its impacts on intrabacterial pH homeostasis.

To address these questions, we developed a dual-imaging approach that allows monitoring of endolysosomal acidification and M. tuberculosis intrabacterial pH homeostasis in real time. Single-cell quantitative analysis shows that M. tuberculosis can maintain its intrabacterial pH independently of the host pH. By live-cell imaging and tracking of M. tuberculosis bacterial populations residing within acidic endolysosomes, we show that long-term residence within acidic microenvironments, i.e., for several hours, is not sufficient to impact M. tuberculosis pH homeostasis. Using this approach, we describe the spatiotemporal dynamics of PZA mode of action within M. tuberculosis-infected human macrophages, showing that phagosomal restriction and host subcellular acidification are crucial for PZA/POA antibacterial efficacy. Finally, by using mycobacterial mutants with different phenotypes and intracellular lifestyles, we define how both host-driven and bacterial factors contribute to PZA efficacy in human macrophages.

## RESULTS

### A live dual-imaging approach to monitor organelle and M. tuberculosis acidification.

**FIG 1:**
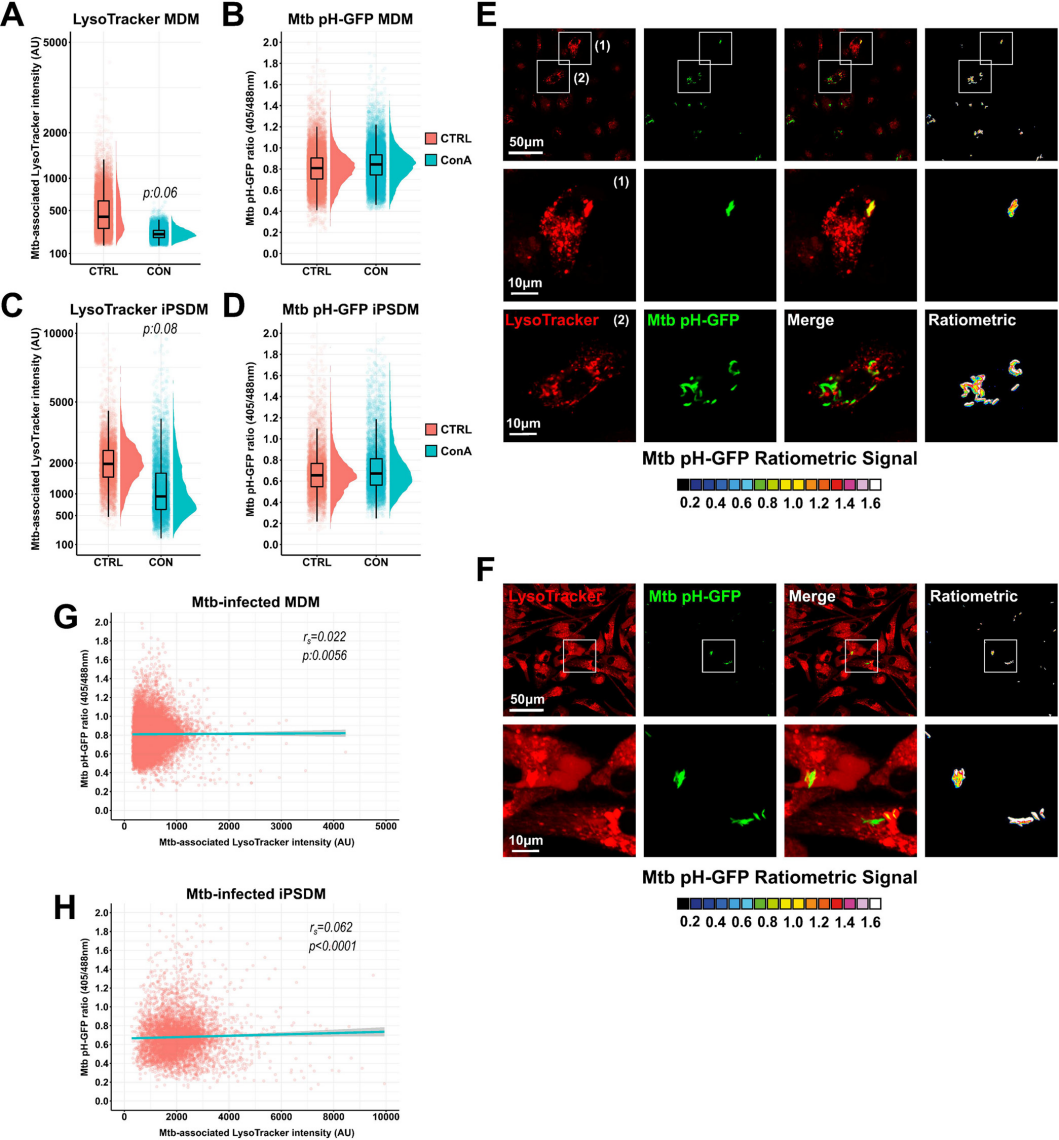
High-content dual imaging of M. tuberculosis intrabacterial pH and host cell intracellular acidification. Human macrophages were infected for 24 h and subsequently treated with ConA or left untreated for an additional 24 h. Cells were then pulsed with 200 nM LysoTracker Red for 30 min before live acquisition was performed using the OPERA Phenix imaging platform. Quantitative analysis of Mtb pH-GFP ratio (405/488 nm) and M. tuberculosis-associated LysoTracker was performed using the Harmony software. (A and C) Quantification of M. tuberculosis-associated LysoTracker mean fluorescence intensities within infected MDM (A) or infected iPSDM (C) in the absence (pink) or presence (cyan) of v-ATPase inhibitor ConA. Results obtained from control (CTRL) or ConA-treated samples are displayed as raincloud plots where black boxplots are overlaid on top of individual raw data and associated with their respective density plots. (B and D) Quantification of Mtb pH-GFP ratio (405/488 nm) within infected MDM (B) or infected iPSDM (D) in the absence (pink) or presence (cyan) of v-ATPase inhibitor ConA. Results obtained from CTRL or ConA-treated samples are displayed as raincloud plots where black boxplots are overlaid on top of individual raw data and associated with their respective density plots. (E and F) Representative micrographs display LysoTracker labeling (red) and Mtb pH-GFP (green). Ratiometric signal was obtained by dividing the fluorescence intensity acquired with excitation/emission channels of 405/510 nm by the one obtained at 488/510 nm. Ratiometric signal is displayed as a 16-color palette ranging from 0 to 1.6 units. Scale bar corresponds to 50 μm. Panel E shows MDM, and panel F shows iPSDM. Regions of interest highlighted by the white rectangles are shown in detail in the bottom panels, respectively. Scale bar corresponds to 10 μm. (G and H) Spearman’s correlation between M. tuberculosis-associated LysoTracker (*x* axis) and Mtb pH-GFP ratio (405/488 nm) (*y* axis) signals in individual bacterial region of interests within infected MDM (G) or infected iPSDM (H). The cyan line shows the linear regression model; the Spearman rank correlation coefficient (*r_s_*) and the corresponding *P* value were calculated by using the ggpubr R package and two-tailed statistical *t* test. Between 5,895 and 15,823 bacterial regions of interest were analyzed per experimental condition. Results are representative of *n* = 2 biologically independent experiments performed at least in two to three technical replicates. Statistical analysis was performed using the Wilcoxon signed-rank test, where untreated control was used as the reference condition.

To concomitantly monitor macrophage organelle and M. tuberculosis acidification status, we developed a high-content dual-imaging approach that allows live-fluorescence microscopy visualization of infected human macrophages, including quantitative measurements of subcellular acidification and M. tuberculosis intrabacterial pH homeostasis. We first generated an M. tuberculosis reporter strain (Mtb pH-GFP) that constitutively produces a ratiometric pH-GFP (green fluorescent protein) indicator to dynamically record intrabacterial pH fluctuations *in vitro* and *in cellulo*, as previously described (11, 31). This reporter possesses two excitation maxima at wavelengths 405 nm and 488 nm, and the ratio of 510-nm fluorescence emissions generated by excitation at these two excitation wavelengths varies as a function of the protonation state of the GFP fluorophore. Therefore, a lower 405-nm/488-nm ratio indicates a lower intrabacterial pH (11, 31). Then, human monocyte-derived macrophages (MDM) and human-induced pluripotent stem cell (iPSC)-derived macrophages (iPSDM) were infected with the reporter strain for 24 h, a time that allows bacteria to adapt intracellularly and localize in multiple niches (15, 18). Infected cells were left untreated or pulsed for an additional 24 h with concanamycin A (ConA), a selective v-ATPase inhibitor that inhibits endolysosomal acidification (43), and further stained with the lysosomotropic fluorescent probe LysoTracker to visualize acidic endolysosomes and determine host subcellular acidification profile. Next, M. tuberculosis-associated LysoTracker intensity and M. tuberculosis intrabacterial pH were analyzed by automated high-content microscopy (see Fig. S1 in the supplemental material). A quantitative analysis in M. tuberculosis-infected MDM (median_CTRL_ = 422.6, IQR_CTRL_ = 334.9, and median_ConA_ = 241.6, IQR_ConA_ = 64.5, respectively) and M. tuberculosis-infected iPSDM (median_CTRL_ = 1,964.2, IQR_CTRL_ = 1,006.0, and median_ConA_ = 929.3, IQR_ConA_ = 1,009.4, respectively) (IQR stands for interquartile range) showed that the median M. tuberculosis-associated LysoTracker intensity was reduced by approximately 2-fold upon ConA treatment (*P* values 0.06 and 0.08, respectively) (Fig. 1A and C), confirming that endolysosomal acidification was impaired (23). On the other hand, a quantitative analysis of M. tuberculosis intrabacterial pH under control or ConA-treated conditions showed that they were similar in both infected MDM (Fig. 1B) and infected iPSDM (Fig. 1D) with absolute median differences that were almost null (Δmedian_pH-GFP_ = 0.035 and 0.016, respectively), suggesting that intracellular acidification does not impact M. tuberculosis intrabacterial pH in human macrophages. To confirm that M. tuberculosis can maintain its intrabacterial pH independently of macrophage pH, we determined the Spearman correlation coefficient between Mtb pH-GFP ratio values and their corresponding associated LysoTracker intensity. There was no positive or negative association with correlation coefficients of *r_s_* = 0.022, *P < *0.01, and *r_s_* = 0.062, *P < *0.0001, in MDM or iPSDM, respectively (Fig. 1E to H). Next, we performed live-image acquisition of M. tuberculosis-infected LysoTracker-stained iPSDM at higher resolution (18, 42). In agreement with the previous findings (Fig. 1G and H), analysis of Spearman’s correlation coefficient did not show any correlation between subcellular acidification profile and M. tuberculosis intrabacterial pH with a value of *r_s_* = −0.19, *P < *0.0001 (Fig. S2A). Altogether, these data support the notion that M. tuberculosis can maintain its own pH when facing a wide range of *in vitro* and *in cellulo* environmental pH (11, 31, 32).

10.1128/mbio.00117-22.1 FIG S1 Analytical pipeline used in this study to perform high-content quantitative analysis of fluorescence microscopy images. (A) Schematic representation of the segmentation and analysis pipeline used in this study to perform quantitative analysis of M. tuberculosis intrabacterial pH and M. tuberculosis-associated LysoTracker intensity by fluorescence microscopy. (B) Representative images of Mtb pH-GFP-infected MDM stained with LysoTracker. Infected cells were infected for 48 h and then pulsed with 200 nM LysoTracker Red for 30 min. Cells were imaged live using the high-content screening platform OPERA Phenix. Micrographs display LysoTracker labeling (red) and Mtb pH-GFP (green). Ratiometric signal was obtained by dividing the fluorescence intensity acquired with excitation/emission channels of 405/510 nm by the one obtained at 488/510 nm. Ratiometric signal is displayed as a 16-color palette ranging from 0 to 1.6 units. Scale bar corresponds to 50 μm. Regions of interest, highlighted by the white rectangles, are shown in detail in the bottom panels, respectively. Scale bar corresponds to 10 μm. The complete procedures are detailed in Materials and Methods. Download FIG S1, PDF file, 0.5 MB. Copyright © 2022 Santucci et al. 2022 Santucci et al. https://creativecommons.org/licenses/by/4.0/ This content is distributed under the terms of the Creative Commons Attribution 4.0 International license.

10.1128/mbio.00117-22.2 FIG S2 Monitoring of M. tuberculosis intrabacterial pH and host cell intracellular acidification by an alternative low-content imaging approach. Human macrophages were infected for 48 h and then pulsed with 200 nM LysoTracker Red for 30 min before live acquisition was performed using a Leica SP5 AOBS laser scanning confocal microscope. Quantitative analysis of Mtb pH-GFP ratio (405/488 nm) and M. tuberculosis-associated LysoTracker was performed using the open-source Fiji software. (A) Spearman’s correlation between M. tuberculosis-associated LysoTracker (*x* axis) and Mtb pH-GFP ratio (405/488 nm) (*y* axis) signals in individual bacterial regions of interest within infected iPSDM. The linear regression is shown as the cyan line; the Spearman rank correlation coefficient (*r_s_*) and the corresponding *P* value were calculated by using the ggpubr R package and two-tailed statistical *t* test. (B) Representative micrographs display LysoTracker labeling (red) and Mtb pH-GFP (green). Ratiometric signal was obtained by dividing the fluorescence intensity acquired with excitation/emission channels of 405/510 nm by the one obtained at 488/510 nm. Ratiometric signal is displayed as a 16-color palette ranging from 0 to 1.6 units. Scale bar corresponds to 50 μm. (C) Regions of interest 1 to 4, highlighted by the white rectangles in panel B, are shown in detail in the bottom panels, respectively. Scale bar corresponds to 10 μm. Results are representative of *n* = 2 biologically independent experiments performed at least in two to three technical replicates. Download FIG S2, PDF file, 0.9 MB. Copyright © 2022 Santucci et al. 2022 Santucci et al. https://creativecommons.org/licenses/by/4.0/ This content is distributed under the terms of the Creative Commons Attribution 4.0 International license.

### M. tuberculosis subcellular localization within acidic compartments and time of residence does not affect bacterial pH homeostasis.

**FIG 2:**
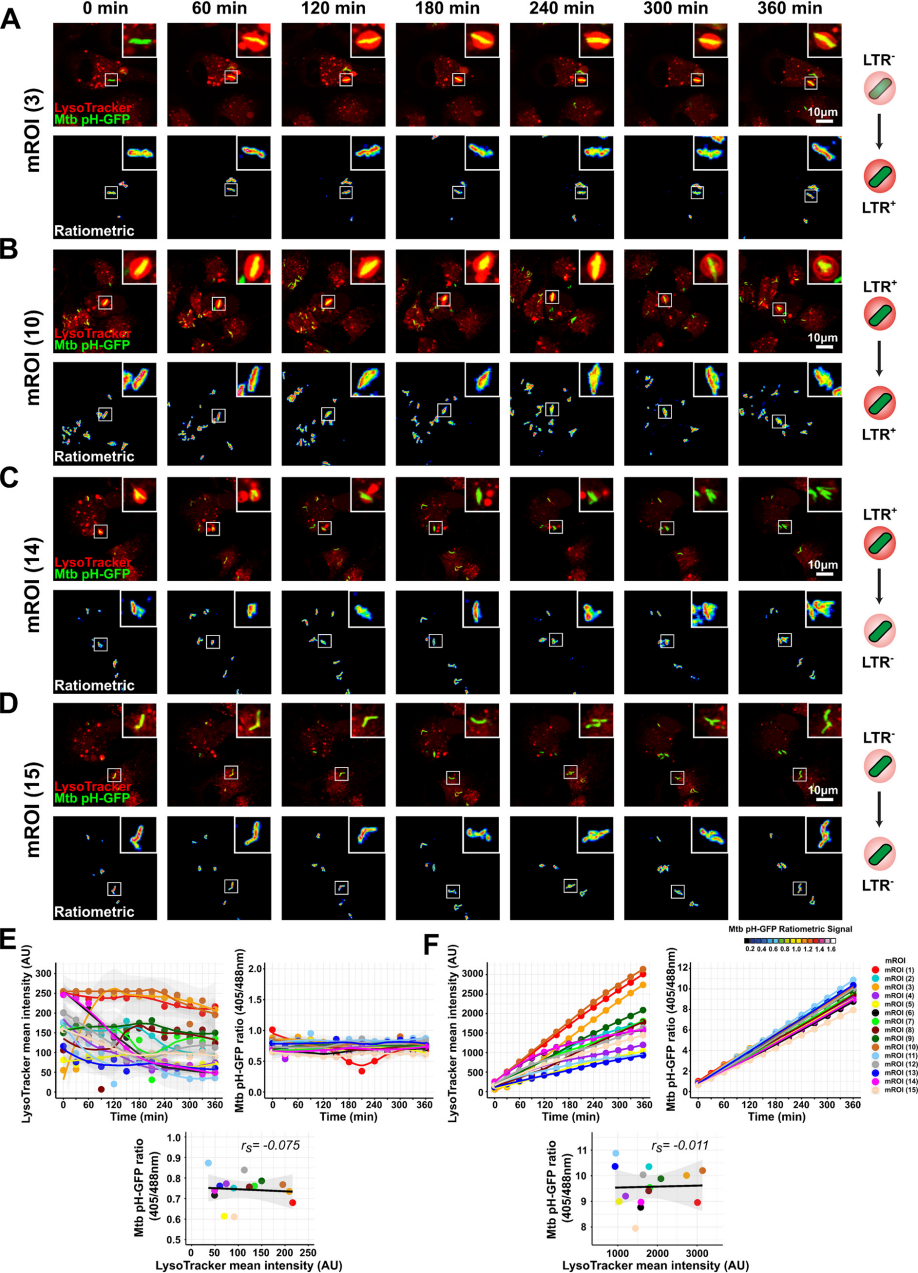
Live-cell imaging of M. tuberculosis pH homeostasis in acidic subcellular compartments. Human iPSDM were infected for 24 h and then pulsed with 200 nM LysoTracker Red for 30 min before live acquisition was performed using a Leica SP5 AOBS laser scanning confocal microscope. Quantitative analysis of Mtb pH-GFP ratio (405/488 nm) and M. tuberculosis-associated LysoTracker was performed using the open-source Fiji software. (A to D) Representative micrographs display LysoTracker labeling (red) and Mtb pH-GFP (green) of 4 distinct mROI (3, 10, 14, and 15, respectively) with different LysoTracker-associated fluorescence patterns along the kinetic. Ratiometric signal was obtained by dividing the fluorescence intensity acquired with excitation/emission channels of 405/510 nm by the one obtained at 488/510 nm. Ratiometric signal is displayed as a 16-color palette ranging from 0 to 1.6 units. Events of interest are highlighted with white squares, and a zoom-in is displayed at the top right corner of each micrograph. Scale bar corresponds to 10 μm. (E) Quantitative analysis of intracellular M. tuberculosis-associated LysoTracker intensity and Mtb pH-GFP profiles of single-tracked mROI over time. Left panel shows the mean LysoTracker intensity associated with Mtb pH-GFP, and the right panel shows their corresponding fluorescence ratio profiles. Bottom panel shows Spearman’s correlation between M. tuberculosis-associated LysoTracker (*x* axis) and Mtb pH-GFP ratio (405/488 nm) (*y* axis) signals from single-tracked mROI at the end of the kinetic (*t*_360 min_). Spearman rank correlation coefficient (*r_s_*) was calculated by using the ggpubr R package. Each color represents one mROI, and the corresponding key is displayed in panel F. (F) Quantitative analysis of cumulative M. tuberculosis-associated LysoTracker intensity and Mtb pH-GFP profiles of single-tracked mROI over time. Left panel shows the cumulative mean LysoTracker intensity associated with Mtb pH-GFP, and the right panel shows their corresponding fluorescence ratio profiles. Bottom panel shows Spearman’s correlation between cumulative M. tuberculosis-associated LysoTracker values (*x* axis) and cumulative Mtb pH-GFP ratio values (405/488 nm) (*y* axis) signals from single-tracked mROI over the kinetic (*t*_360 min_). Spearman rank correlation coefficient (*r_s_*) was calculated by using the ggpubr R package. Each color represents one mROI, and the corresponding key is displayed on the right of the panel. Results are from *n* = 15 individually tracked mROI.

To complement our live-snapshot imaging approach and capture the dynamic nature of these transient events, we performed live imaging at low content/high resolution and tracked individual mycobacterial regions of interest (mROI) to define whether the time of residency within LysoTracker-positive compartments impacts M. tuberculosis intrabacterial pH. Live-cell imaging of M. tuberculosis-infected iPSDM was performed over a 6-h period with 30-min intervals to minimize photobleaching and/or phototoxicity. Monitoring and tracking of mROI revealed at least 4 different phenotypic profiles: (i) LysoTracker^(−)^ M. tuberculosis that became LysoTracker^(+)^ (Fig. 2A), (ii) LysoTracker^(+)^ M. tuberculosis that remained LysoTracker^(+)^ (Fig. 2B), (iii) LysoTracker^(+)^ M. tuberculosis that became LysoTracker^(−)^ (Fig. 2C), and (iv) LysoTracker^(−)^ M. tuberculosis that remained LysoTracker^(−)^ (Fig. 2D). Next, we analyzed the dynamics of M. tuberculosis-associated LysoTracker intensity and pH-GFP ratio (Fig. 2E) as previously described (18, 42). We observed that over time the association with LysoTracker was very heterogenous and that Mtb pH-GFP ratio did not significantly change, with no correlation between LysoTracker association and Mtb pH-GFP ratio (Fig. 2E). To exclude the possibility that prolonged exposure to low pH within an acidified compartment impacts M. tuberculosis pH homeostasis, we analyzed the cumulative values of M. tuberculosis-associated LysoTracker fluorescence intensity over time to include total LysoTracker intensity association during 360 min and the corresponding Mtb pH-GFP ratios (Fig. 2F), thus giving a quantitative profile of LysoTracker intensity faced by multiple mROI over the entire time course. In agreement with the previous analysis, despite heterogenous accumulation of LysoTracker with M. tuberculosis, there were no significant changes in M. tuberculosis pH homeostasis (Fig. 2F). Determination of Spearman’s correlation coefficients at the end time point did not show positive or negative association between subcellular acidification profile and M. tuberculosis intrabacterial pH with a value of *r_s_* = −0.075 and *r_s_* = −0.011 for the two different analyses, respectively (Fig. 2E and F). We concluded that M. tuberculosis is able to maintain its intracellular pH even when facing fluctuating acidic intracellular environments (11, 31, 32).

### Spatiotemporal analysis of PZA-mediated M. tuberculosis intrabacterial pH homeostasis disruption *in cellulo*.

**FIG 3:**
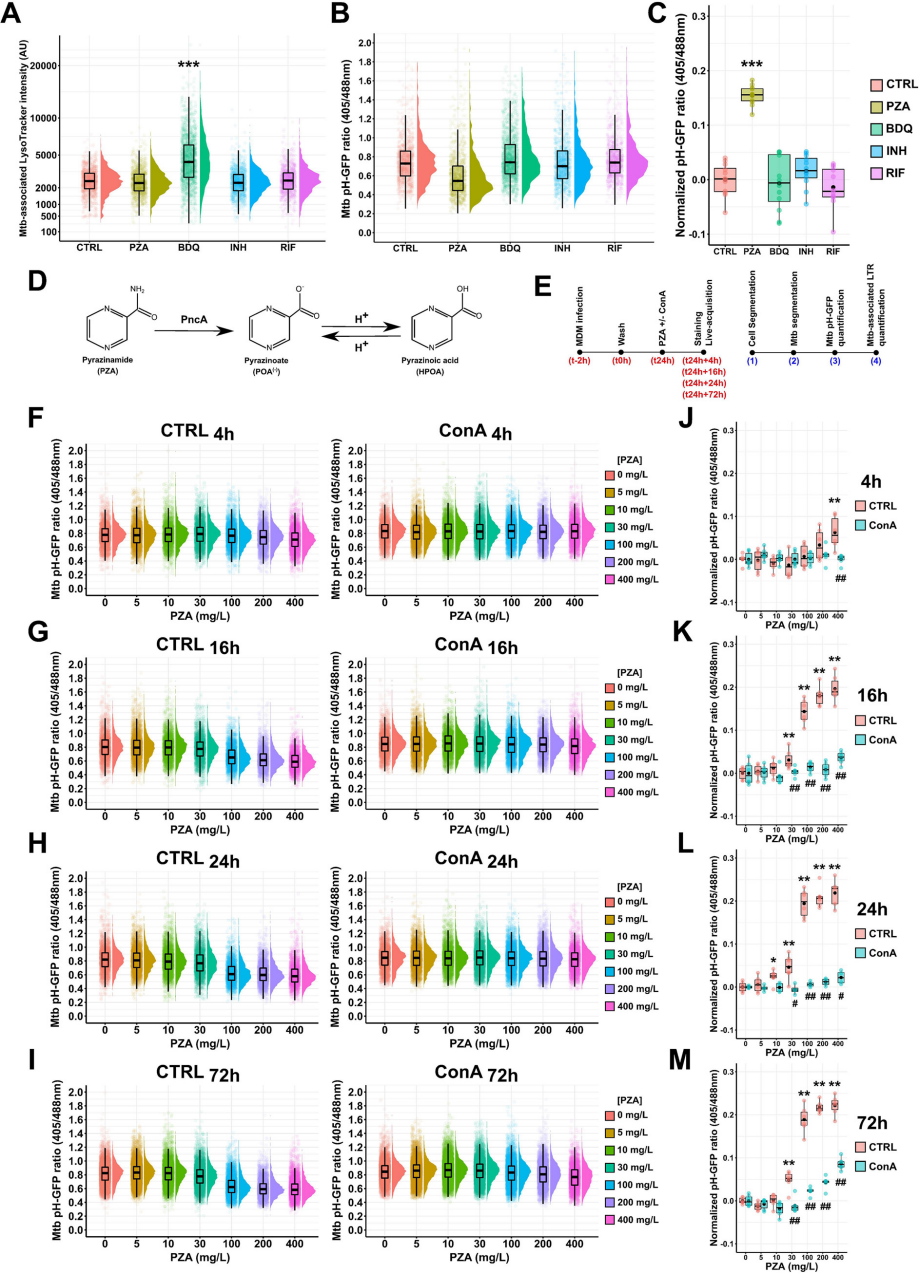
Spatiotemporal analysis of PZA-mediated M. tuberculosis intrabacterial pH homeostasis disruption *in cellulo.* (A to C) Human macrophages were infected with Mtb pH-GFP for 24 h and subsequently treated with either 100 mg/L of PZA, 2.5 mg/L of BDQ, 5 mg/L of INH, or 5 mg/L of RIF or left untreated for 24 h. Cells were then pulsed with 200 nM LysoTracker Red for 30 min before live acquisition was performed using the OPERA Phenix imaging platform. (A and B) Quantification of M. tuberculosis-associated LysoTracker mean intensity (A) or Mtb pH-GFP ratio (405/488 nm) (B) within infected MDM treated with different antibiotics for 24 h. Results are displayed as raincloud plots where black boxplots are overlaid on top of individual raw data and associated with their respective density plots. Between 1,102 and 1,658 bacterial regions of interest were analyzed per experimental condition. (C) Determination of absolute changes in Mtb pH-GFP ratio (405/488 nm) upon various antibiotic treatments. Mean pH-GFP ratio of each antibiotic treatment was subtracted from the untreated control 24 h posttreatment to obtain an absolute value reflecting antibiotic-mediated pH disruption normalized to the control. Determination of normalized pH-GFP ratio was performed for each condition, and results are displayed as boxplots with individual replicate data. Black dots were added to highlight the mean of each condition. Each color represents a specific antibiotic or the control condition. Results are from *n* = 2 biologically independent experiments performed at least in two to four technical replicates. Statistical analysis was performed using Wilcoxon signed-rank test, where untreated control was used as the reference condition (*, *P* ≤ 0.05; **, *P* ≤ 0.01; ***, *P* ≤ 0.001). (D) Chemical structures of PZA, POA^(−)^, and HPOA. Conversion of the prodrug PZA into POA^(−)^ is mediated by the bacterial pyrazinamidase PncA, and transition of POA^(−)^ into HPOA is driven by proton availability. (E) Schematic representation of the experimental procedure followed to perform MDM infection, staining, and fluorescence microscopy imaging. (F to M) Human macrophages were infected for 24 h and subsequently treated with increasing concentrations of PZA ranging from 0 to 400 mg/L in the absence or presence of ConA for 4 h, 16 h, 24 h, or 72 h. Cells were then pulsed with 200 nM LysoTracker Red for 30 min before live acquisition was performed using the OPERA Phenix imaging platform. (F to I) Quantification of Mtb pH-GFP ratio (405/488 nm) within infected MDM treated with increasing concentrations of PZA ranging from 0 to 400 mg/L in the absence or presence of v-ATPase inhibitor ConA at 4 h, 16 h, 24 h, or 72 h posttreatment. Results are displayed as raincloud plots where black boxplots are overlaid on top of individual raw data and associated with their respective density plots. Each color represents a specific PZA concentration. Between 2,164 and 8,813 bacterial regions of interest were analyzed per experimental condition. (J to M) Determination of absolute changes in Mtb pH-GFP ratio (405/488 nm) upon PZA treatment. Mean pH-GFP ratio of each antibiotic treatment was subtracted from the PZA-untreated control to obtain an absolute value reflecting antibiotic-mediated pH disruption normalized to its respective control in the presence or absence of ConA at 4 h, 16 h, 24 h, or 72 h posttreatment. Determination of normalized pH-GFP ratio was performed in the absence (pink) or presence (cyan) of v-ATPase inhibitor ConA. Results are displayed as boxplots with individual data. Black dots were added to highlight the mean of each condition. Results are from *n* = 2 biologically independent experiments performed at least in two to three technical replicates. Statistical analysis was performed using Wilcoxon signed-rank test, where PZA effect on M. tuberculosis intrabacterial pH was assessed against the untreated control (*, *P* ≤ 0.05; **, *P* ≤ 0.01; ***, *P* ≤ 0.001) and ConA effect toward PZA was assessed by comparing each concentration with its respective untreated control as reference condition (#, *P* ≤ 0.05; ##, *P* ≤ 0.01; ###, *P* ≤ 0.001).

The molecular mechanism(s) of PZA action has been mostly experimentally tested within *in vitro* cell-free media, and it is unknown whether PZA/POA molecules are able to disrupt M. tuberculosis intrabacterial pH homeostasis *in cellulo*. To address this question, human macrophages were infected with Mtb pH-GFP for 24 h and subsequently treated with PZA, bedaquiline (BDQ), INH, or RIF or left untreated for an additional 24 h before being stained with LysoTracker and live imaged. Quantitative analysis of M. tuberculosis-associated LysoTracker intensity revealed that BDQ treatment was the only condition impacting M. tuberculosis-associated LysoTracker intensity profile as previously reported (median_BDQ_ = 4,252.1, IQR_BDQ_ = 3,369.3, and median_CTRL_ = 2,502.2, IQR_CTRL_ = 1,221.3, respectively) (Fig. 3A) (44). However, despite its proposed ionophore activity *in vitro* (45) and potent activity of enhancing the intracellular acidification processes (44), such effects were not sufficient to overcome bacterial regulation of cytosolic pH, as we did not observe changes in M. tuberculosis intrabacterial pH in the presence of BDQ (mean normalized pH-GFP ratio of −0.006; *P* value = 0.887) (Fig. 3B and C). These results are in line with previous studies that have questioned the ability of BDQ and diarylquinolines to alter M. tuberculosis intrabacterial pH and the significance of this process in the antibacterial activity of these compounds (46, 47). Similar results were obtained in the presence of INH and RIF where Mtb pH-GFP ratios were similar to the untreated control condition (mean normalized pH-GFP ratio of 0.016, *P* value = 0.160, and ratio of −0.014, *P* value = 0.319, respectively) (Fig. 3B and C). Strikingly, from the four different antibiotics tested, PZA was the only one able to induce changes in Mtb pH-GFP ratio, providing evidence that PZA displays intrabacterial pH-disruptive activity in M. tuberculosis-infected human macrophages (mean normalized pH-GFP ratio of 0.155; *P* value ≤ 0.001) (Fig. 3C). PZA/POA molecules require endolysosomal acidification to accumulate inside M. tuberculosis and display antimicrobial efficacy (23). We hypothesized that this process is likely resulting from the conversion of POA^(−)^ into its protonated form HPOA within acidic host microenvironments (Fig. 3D). To investigate whether PZA/POA-mediated intrabacterial pH homeostasis disruption *in cellulo* requires endolysosomal acidification, M. tuberculosis-infected MDM were treated with increasing concentration of PZA ranging from 0 to 400 mg/L in the presence or absence of the v-ATPase inhibitor ConA, and both host and bacterial acidification profiles were monitored at 4 h, 16 h, 24 h, and 72 h posttreatment (Fig. 3E) using our live dual-imaging approach. Quantitative analysis of Mtb pH-GFP fluorescence profiles at the indicated time points in untreated control cells confirmed that M. tuberculosis can stably maintain intrabacterial pH through the course of the infection (Fig. 3F to I). Treatment of infected human macrophages with PZA was able to decrease M. tuberculosis intrabacterial pH in a time- and concentration-dependent manner (Fig. 3F to I). After 4 h of treatment, only 400 mg/L of PZA showed a detectable effect on M. tuberculosis intrabacterial pH (mean normalized pH-GFP ratio of 0.0625; *P* value ≤ 0.01) (Fig. 3F). After 16 h, 24 h, and 72 h, PZA concentrations ranging from 30 mg/L to 400 mg/L significantly disrupted bacterial pH homeostasis (Fig. 3G to I). The absolute changes in Mtb pH-GFP ratio relative to the control condition confirmed a time- and concentration-dependent effect of PZA on M. tuberculosis intrabacterial pH (Fig. 3J to M). Importantly, PZA treatment did not induce rerouting of M. tuberculosis into endolysosomal compartments (Fig. S3), ruling out a concentration-dependent effect toward pH-GFP ratio due to excessive lysosomal delivery. We also noticed that the LysoTracker intensity profile decreased over time independently of the infection, suggesting that human primary macrophages display optimal lysosomal activity for a limited amount of time during their *in vitro* life span (Fig. S4). ConA cotreatment with increasing concentrations of PZA resulted in an almost complete loss of PZA M. tuberculosis pH-disruptive function (Fig. 3J to M), suggesting that functional acidification of the M. tuberculosis phagosome is a prerequisite for PZA-mediated pH disruption in intracellular M. tuberculosis. Quantitative correlative analysis of Mtb pH-GFP ratio values with associated LysoTracker intensity at increasing PZA concentrations did not show a direct association, with Spearman’s correlation coefficients *r_s_* between −0.3 and 0.3 (Fig. S5). These data highlight that PZA-mediated pH homeostasis disruption within acidic environments is a dynamic process.

10.1128/mbio.00117-22.3 FIG S3 Temporal dynamics of M. tuberculosis-associated LysoTracker intensity upon PZA treatment *in cellulo.* Human macrophages were infected for 24 h and subsequently treated with increasing concentrations of PZA ranging from 0 to 400 mg/L in the absence or presence of ConA for 4 h, 16 h, 24 h, or 72 h. Cells were then pulsed with 200 nM LysoTracker Red for 30 min before live acquisition was performed using the OPERA Phenix imaging platform. (A to D) Quantification of M. tuberculosis-associated LysoTracker mean intensity within infected MDM treated with increasing concentrations of PZA ranging from 0 to 400 mg/L in the absence or presence of v-ATPase inhibitor ConA at 4 h, 16 h, 24 h, or 72 h posttreatment. Results are displayed as raincloud plots where black boxplots are overlaid on top of individual raw data and associated with their respective density plots. Each color represents a specific PZA concentration. Results are from *n* = 2 biologically independent experiments performed at least in two to three technical replicates. Download FIG S3, PDF file, 0.9 MB. Copyright © 2022 Santucci et al. 2022 Santucci et al. https://creativecommons.org/licenses/by/4.0/ This content is distributed under the terms of the Creative Commons Attribution 4.0 International license.

10.1128/mbio.00117-22.4 FIG S4 Temporal dynamics of LysoTracker intensity within infected and uninfected human macrophages. Human macrophages were infected with Mtb pH-GFP for 24 h or left uninfected for an additional 4 h, 16 h, 24 h, or 72 h. Cells were then pulsed with 200 nM LysoTracker Red for 30 min before live acquisition was performed using the OPERA Phenix imaging platform. (A and B) Quantification of LysoTracker mean intensity within uninfected MDM (A) or M. tuberculosis-infected MDM (B) cellular region was determined at 4 h, 16 h, 24 h, or 72 h posttreatment. Results are displayed as boxplots with individuals’ data. Black dots were added to highlight the mean of each condition. Each color represents a specific time point. (C) Representative micrographs display LysoTracker labeling (red) and Mtb pH-GFP (green). Scale bar corresponds to 50 μm. Results are from *n* = 2 biologically independent experiments performed at least in two to three technical replicates. Download FIG S4, PDF file, 0.5 MB. Copyright © 2022 Santucci et al. 2022 Santucci et al. https://creativecommons.org/licenses/by/4.0/ This content is distributed under the terms of the Creative Commons Attribution 4.0 International license.

10.1128/mbio.00117-22.5 FIG S5 PZA-mediated disruption of M. tuberculosis intrabacterial pH does not directly correlate with M. tuberculosis-associated LysoTracker intensity within human macrophages. Human macrophages were infected for 24 h and subsequently treated with increasing concentration of PZA ranging from 0 to 400 mg/L for 4 h, 16 h, 24 h, or 72 h. Cells were then pulsed with 200 nM LysoTracker Red for 30 min before live acquisition was performed using the OPERA Phenix imaging platform. (A to D) Spearman’s correlation between M. tuberculosis-associated LysoTracker (*x* axis) and Mtb pH-GFP ratio (405/488 nm) (*y* axis) signals in individual bacterial regions of interest within infected MDM at 4 h, 16 h, 24 h, or 72 h posttreatment, respectively. Spearman rank correlation coefficient (*r_s_*), shown as a regression line, was calculated by using the ggpubr R package. Each color represents a specific PZA concentration. Results are from *n* = 2 biologically independent experiments performed at least in two to three technical replicates. Download FIG S5, PDF file, 0.9 MB. Copyright © 2022 Santucci et al. 2022 Santucci et al. https://creativecommons.org/licenses/by/4.0/ This content is distributed under the terms of the Creative Commons Attribution 4.0 International license.

### Endolysosomal acidification and pH-disruptive activity of PZA contribute to M. tuberculosis restriction in human macrophages.

**FIG 4:**
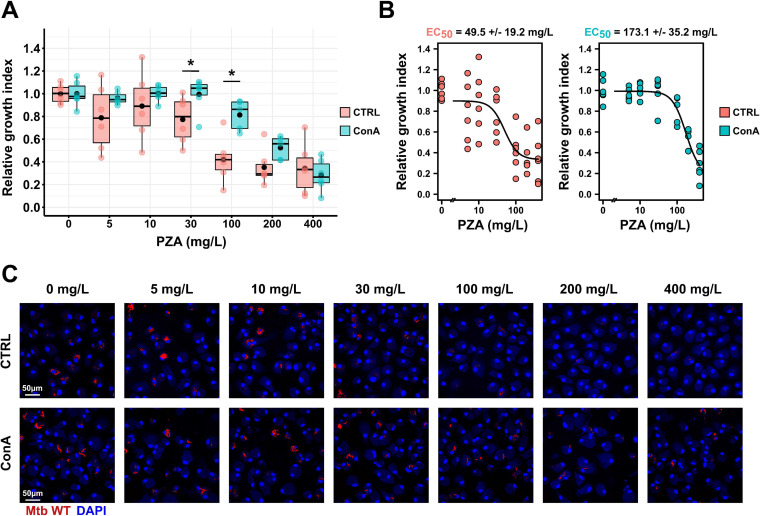
PZA-mediated growth inhibition requires endolysosomal acidification within human macrophages. Human macrophages were infected with M. tuberculosis WT E2-Crimson for 24 h and subsequently treated with increasing concentrations of PZA ranging from 0 to 400 mg/L in the absence or presence of ConA for 72 h. (A) Quantitative analysis of E2-Crimson M. tuberculosis WT replication at the single-cell level within MDM treated with increasing concentrations of PZA in the absence or presence of ConA. Normalization was done to the mean M. tuberculosis area per cell pretreatment (*t*_24h_ postinfection), and the control condition without PZA was used as reference corresponding to 100% growth. Determination of relative growth index was performed in the absence (pink) or presence (cyan) of v-ATPase inhibitor ConA. Results are displayed as boxplots with individual replicate data. Black dots were added to highlight the mean of each condition. Between 1,185 and 1,925 M. tuberculosis-infected MDM were analyzed by high-content single-cell microscopy, and results are representative of *n* = 2 biologically independent experiments performed at least in two to three technical replicates. Statistical analysis was performed using Wilcoxon signed-rank test, where untreated control was used as the reference condition (*, *P* ≤ 0.05; **, *P* ≤ 0.01; ***, *P* ≤ 0.001). (B) Determination of PZA EC_50_ in the absence or presence of ConA by performing a 4-parameter nonlinear logistic regression of the data displayed in panel A. (C) Representative confocal fluorescence images of M. tuberculosis WT-infected MDM infected for 24 h and further treated for 72 h with increasing concentrations of PZA. Magnifications display nuclear staining (blue) and M. tuberculosis producing E2-Crimson (red). Scale bar corresponds to 50 μm. Micrographs are representative of 2 independent experiments.

We next hypothesized that functional host intracellular acidification and PZA-mediated pH decrease are required for mycobacterial growth inhibition. In order to test this hypothesis, we quantified M. tuberculosis intracellular replication at the single-cell level in the presence of increasing concentration of PZA in both control and ConA-treated cells (Fig. 4A). Results from dose-response analysis showed that functional endolysosomal acidification, required for PZA-mediated pH disruption, is also required for optimal PZA efficacy and M. tuberculosis growth restriction over the course of infection (Fig. 4A). The determination of PZA half-maximal effective concentration (EC_50_) using a four-parameter logistic nonlinear regression model showed that ConA cotreatment increased, by approximately 3.5 times, the amount of antibiotic required to efficiently inhibit 50% of M. tuberculosis growth *in cellulo* (49.5 ± 19.2 mg/L and 173.1 ± 35.2 mg/L, respectively) (Fig. 4A and B). Surprisingly, when using 200 mg/L or 400 mg/L of PZA, no significant differences between the control and the ConA-treated cells regarding M. tuberculosis growth were observed (Fig. 4A and B). We hypothesized that PZA at higher concentrations might be able to diffuse evenly within M. tuberculosis-infected cells and reach the entire population contained within distinct subcellular compartments including membrane-bound and cytosolic bacteria. This also suggests that at very high concentrations, the contribution of external acidic pH required for PZA/POA to display inhibitory effects might be limited. The results obtained in this experimental setting agree with our previous observations showing that the use of v-ATPase inhibitors is able to counteract PZA/POA-mediated growth inhibition by impairing POA accumulation within the bacteria (23). These experiments were also performed in another human macrophage model using iPSDM (Fig. S6). Notably, in iPSDM antagonistic effects between PZA and ConA were also observed; however, the phenotypes were less pronounced than in M. tuberculosis-infected MDM, suggesting that iPSDM and MDM might have different intracellular pH homeostatic processes. Altogether, these findings support the proposed pH-dependent mode of action of PZA, in which endolysosomal acidification is a prerequisite and driver of PZA/POA-mediated pH homeostasis disruption, which controls bacterial growth in human macrophages.

10.1128/mbio.00117-22.6 FIG S6 PZA triggers M. tuberculosis intrabacterial pH disruption in GM-CSF-derived iPSDM. Human macrophages were infected for 24 h and subsequently treated with increasing concentrations of PZA ranging from 0 to 400 mg/L in the absence or presence of ConA for 24 h. Cells were then pulsed with 200 nM LysoTracker Red for 30 min before live acquisition was performed using the OPERA Phenix imaging platform. (A) Quantification of Mtb pH-GFP ratio (405/488 nm) within infected iPSDM treated with increasing concentrations of PZA ranging from 0 to 400 mg/L in the absence or presence of v-ATPase inhibitor ConA at 24 h posttreatment. Results are displayed as raincloud plots where black boxplots are overlaid on top of individual raw data and associated with their respective density plots. Each color represents a specific PZA concentration. (B) Determination of absolute changes in Mtb pH-GFP ratio (405/488 nm) upon PZA treatment. Mean pH-GFP ratio of each condition was subtracted from the PZA-untreated condition in the absence or presence of ConA at 24 h posttreatment to obtained an absolute value reflecting PZA-mediated pH disruption normalized to PZA-free conditions. Determination of normalized pH-GFP ratio was performed in the absence (pink) or presence (cyan) of v-ATPase inhibitor ConA. Results are displayed as boxplots with individuals’ raw data. Black dots were added to highlight the mean of each condition. Results are representative of *n* = 2 biologically independent experiments performed at least in two to three technical replicates. (C) Quantitative analysis of E2-Crimson Mtb WT replication at the single-cell level within iPSDM treated with increasing concentrations of PZA in the absence or presence of ConA. Normalization was done to the mean M. tuberculosis area per cell pretreatment (*t*_24h_ postinfection), and the control condition without PZA was used as reference corresponding to 100% growth. Determination of relative growth index was performed in the absence (pink) or presence (cyan) of v-ATPase inhibitor ConA. Results are displayed as boxplots with individuals’ raw data. Black dots were added to highlight the mean of each condition. (D) Determination of PZA EC_50_ in the absence or presence of ConA by performing a four-parameter nonlinear logistic regression of the data displayed in panel A. Results are representative of *n* = 2 biologically independent experiments performed at least in two to three technical replicates. (E) Representative confocal fluorescence images of Mtb WT-infected iPSDM for 24 h and further treated for 72 h with increasing concentrations of PZA. Magnifications display nuclear staining (blue) and M. tuberculosis producing E2-Crimson (red). Scale bar corresponds to 50 μm. Micrographs are representative of 2 independent experiments. Download FIG S6, PDF file, 1.0 MB. Copyright © 2022 Santucci et al. 2022 Santucci et al. https://creativecommons.org/licenses/by/4.0/ This content is distributed under the terms of the Creative Commons Attribution 4.0 International license.

### M. tuberculosis mutants with different subcellular localizations show distinct PZA susceptibility profiles *in cellulo*.

**FIG 5:**
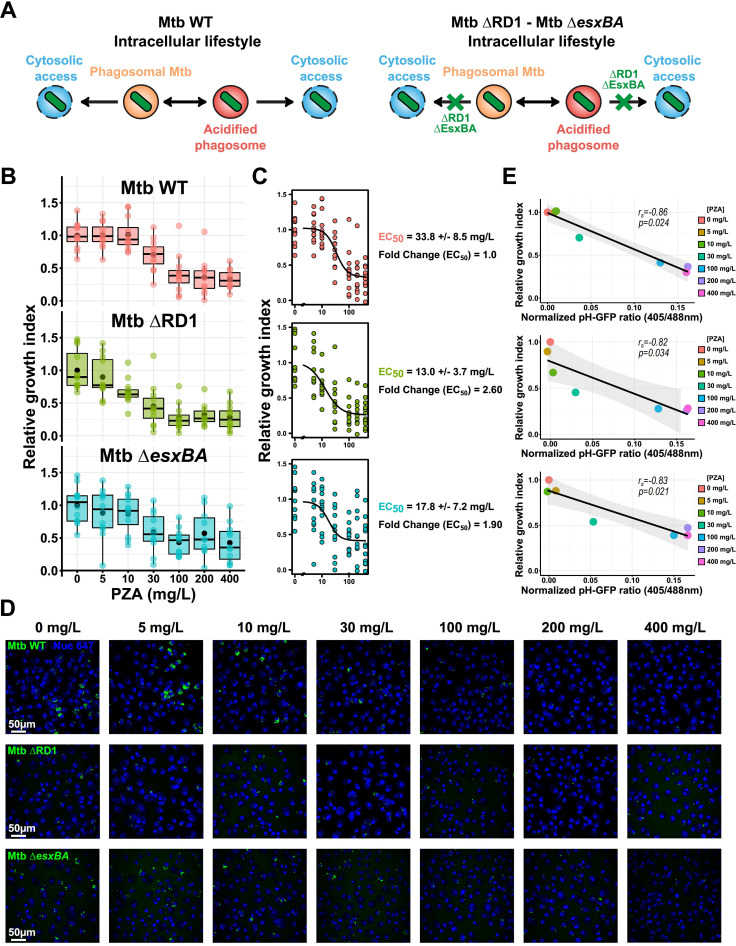
RD1- and EsxBA-mediated endolysosomal damage partially protects M. tuberculosis against PZA activity *in cellulo.* Human macrophages were infected with Mtb WT, Mtb ΔRD1, or Mtb Δ*esxBA* for 24 h and subsequently treated with increasing concentrations of PZA ranging from 0 to 400 mg/L for 72 h. (A) A schematic representation of Mtb WT, Mtb ΔRD1, and Mtb Δ*esxBA* intracellular lifestyles. The contribution of RD1 and EsxBA virulence factors in membrane damage and cytosolic access is highlighted in the right panel. (B) Quantitative analysis of fluorescent M. tuberculosis strains replication at the single-cell level within MDM treated with increasing concentrations of PZA. Normalization was done to the mean M. tuberculosis area per cell pretreatment (*t*_24h_ postinfection), and the control condition without PZA was used as reference corresponding to 100% growth. Results are displayed as boxplots with individual replicate data. Black dots were added to highlight the mean of each condition. Between 3,880 and 10,874 M. tuberculosis-infected MDM were analyzed by high-content single-cell microscopy, and results are representative of *n* = 4 biologically independent experiments performed at least in two to three technical replicates. (C) Determination of PZA EC_50_ for the different M. tuberculosis strains by performing a 4-parameter nonlinear logistic regression of the data displayed in panel B. (D) Representative confocal fluorescence images of Mtb WT-, Mtb ΔRD1-, or Mtb Δ*esxBA*-infected MDM infected for 24 h and further treated for 72 h with increasing concentrations of PZA. Magnifications display nuclear staining (blue) and fluorescent M. tuberculosis (green). Scale bar corresponds to 50 μm. Micrographs are representative of 4 independent experiments. (E) Spearman’s correlation between normalized Mtb pH-GFP ratio (405/488 nm) at 24 h posttreatment (*x* axis) and M. tuberculosis relative growth index (*y* axis) within infected MDM at 72 h posttreatment. Results from Mtb WT, Mtb ΔRD1, and Mtb Δ*esxBA* are shown from the top panel to the bottom panel, respectively. The black line shows the linear regression model; the Spearman rank correlation coefficient (*r_s_*) and the corresponding *P* value were calculated by using the ggpubr R package and two-tailed statistical *t* test. Results are from *n* = 2 or *n* = 4 biologically independent experiments performed at least in two to three technical replicates.

To define how intracellular localization contributes to PZA/POA antibacterial efficacy, we assessed PZA-mediated pH homeostasis disruption and growth inhibition toward multiple M. tuberculosis mutants with distinct intracellular lifestyles (Fig. 5A). Mtb WT (wild type) harboring a functional ESX-1 secretion system was used as the reference strain, and Mtb ΔRD1 lacking a functional ESX-1 machinery was used as a phagosome-restricted strain. We also included another phagosome-restricted mutant, Mtb Δ*esxBA*, which lacks only the two major ESX-1 effectors EsxA and EsxB (also known as ESAT-6 and CFP-10). A relative growth index was quantified for each strain, and a dose-response analysis using a four-parameter logistic nonlinear regression model was performed to determine PZA EC_50_ toward each strain (Fig. 5B and C). Results obtained after curve fitting showed that EC_50_ toward the reference strain Mtb WT was 33.8 ± 8.5 mg/L. Both Mtb ΔRD1 and Mtb Δ*esxBA* displayed increased susceptibility to PZA with EC_50_ values of 13.0 ± 3.7 and 17.8 ± 7.2 mg/L, respectively. Determination of EC_50_ values further highlighted the increase in susceptibility of strains unable to damage the endolysosomal membrane and access host cytosol with a 2.60- and 1.90-fold increase for Mtb ΔRD1 and Mtb Δ*esxBA*, respectively, compared to the WT reference strain (Fig. 5C). Thus, a functional ESX-1 secretion system is protective against PZA/POA activity in M. tuberculosis-infected macrophages, potentially through facilitating membrane damage and cytosolic access. Previous work showed that disruption of M. tuberculosis intrabacterial pH homeostasis caused by pharmacological inhibitors directly correlated with a mycobactericidal effect (31). We investigated whether a PZA-mediated pH decrease was correlated with intracellular growth defects. Relative growth index values were plotted as a function of normalized pH-GFP ratios at each PZA concentration, and Spearman’s correlation coefficients were determined for each strain (Fig. 5E). Notably, PZA-mediated pH homeostasis disruption strongly correlated with the intracellular replication defect (*r_s_* values ranging from −0.82 to −0.86), suggesting that PZA-mediated intrabacterial pH disruption is an important factor in its antibacterial activity (Fig. 5E).

### PZA-mediated intrabacterial pH disruption and growth inhibition *in cellulo* require POA conversion by functional PncA.

**FIG 6:**
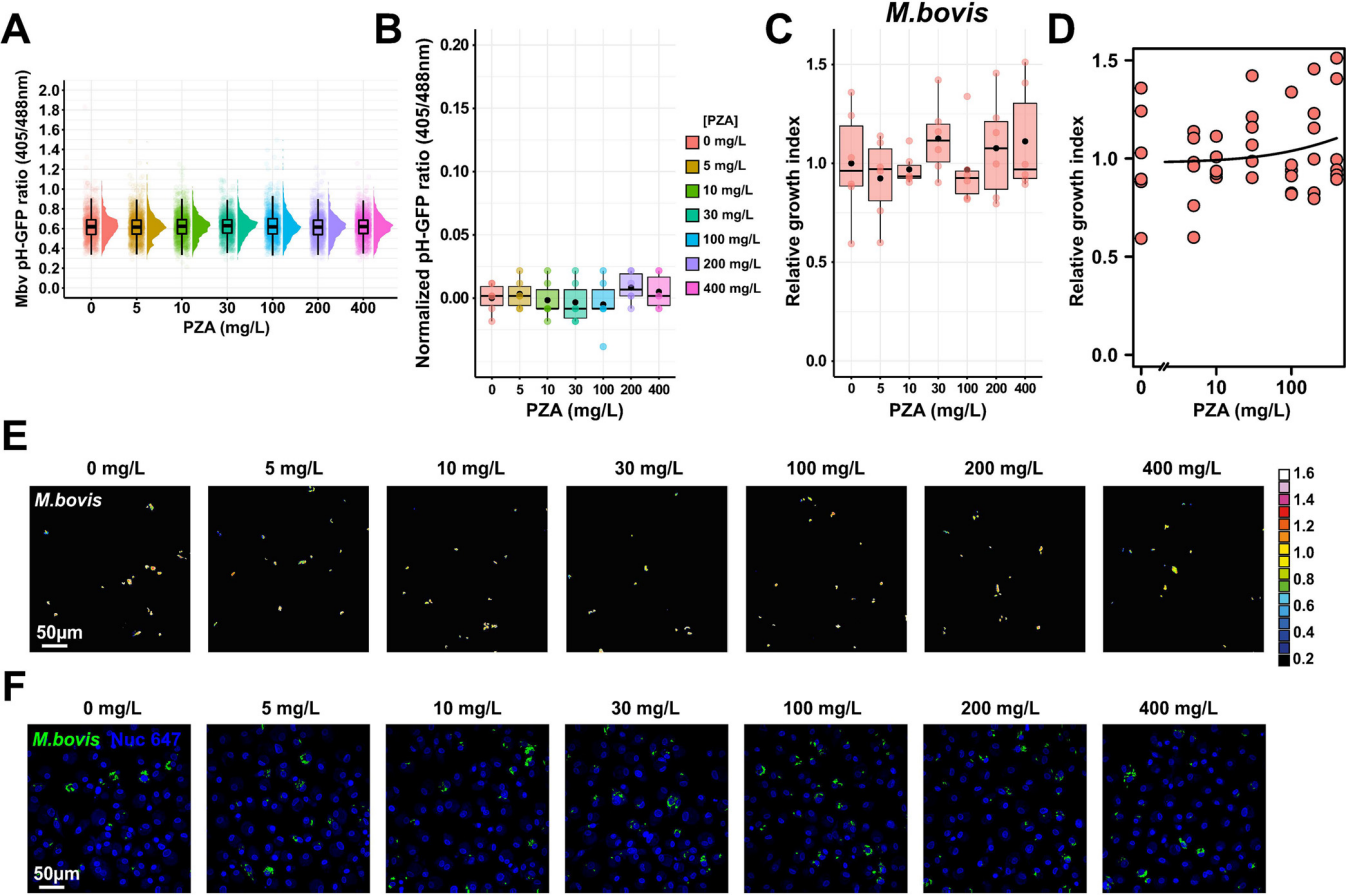
PZA-mediated intrabacterial pH homeostasis disruption and growth inhibition *in cellulo* require functional PncA. Human macrophages were infected with Mbv pH-GFP for 24 h and subsequently treated with increasing concentrations of PZA ranging from 0 to 400 mg/L for 24 h. Cells were then pulsed with 200 nM LysoTracker Red for 30 min before live acquisition was performed using the OPERA Phenix imaging platform. (A) Quantification of Mbv pH-GFP ratio (405/488 nm) within infected MDM treated with increasing concentrations of PZA ranging from 0 to 400 mg/L at 24 h posttreatment. Results are displayed as raincloud plots where black boxplots are overlaid on top of individual replicate data and associated with their respective density plots. Each color represents a specific PZA concentration. (B) Determination of absolute changes in Mbv pH-GFP ratio (405/488 nm) upon PZA treatment. Determination of absolute changes in Mbv pH-GFP ratio (405/488 nm) upon various antibiotic treatments. Mean pH-GFP ratio of each antibiotic treatment was subtracted from the untreated control 24 h posttreatment to obtain an absolute value reflecting antibiotic-mediated pH disruption normalized to the control. Results are displayed as boxplots with individual replicate data. Black dots were added to highlight the mean of each condition. (C and D) Human macrophages were infected with fluorescent M. bovis for 24 h and subsequently treated with increasing concentrations of PZA ranging from 0 to 400 mg/L for 72 h. (C) Quantitative analysis of fluorescent M. bovis strain replication at the single-cell level within MDM treated with increasing concentrations of PZA. Normalization was done to the mean M. bovis area per cell pretreatment (*t*_24h_ postinfection), and the control condition without PZA was used as reference corresponding to 100% growth. Results are displayed as boxplots with individuals’ raw data. Black dots were added to highlight the mean of each condition. Between 4,072 and 4,628 M. bovis-infected MDM were analyzed by high-content single-cell microscopy, and results are representative of *n* = 2 biologically independent experiments performed at least in two to three technical replicates. (D) Determination of PZA EC_50_ for the M. bovis strain by performing a 4-parameter nonlinear logistic regression of the data displayed in panel C. (E) Representative fluorescence ratiometric images of M. bovis-infected MDM infected for 24 h and further treated for 24 h with increasing concentrations of PZA. Ratiometric signal is displayed as a 16-color palette ranging from 0 to 1.6 units. Scale bar corresponds to 50 μm. Micrographs are representative of 2 independent experiments. (F) Representative confocal fluorescence images of M. bovis-infected MDM infected for 24 h and further treated for 72 h with increasing concentrations of PZA. Magnifications display nuclear staining (blue) and fluorescent M. bovis (green). Scale bar corresponds to 50 μm. Micrographs are representative of 2 independent experiments.

Finally, we sought to understand whether PZA conversion to POA was essential to display its pH-disruptive property and antibacterial capacity. To answer this question, we used the bovine TB agent and zoonotic pathogen, Mycobacterium bovis. As a member of the Mycobacterium tuberculosis complex, M. bovis was chosen due to its ability to replicate within human macrophages (48) and its well-characterized intrinsic resistance toward PZA (49, 50). Indeed, M. bovis harbors a point mutation within its *pncA* gene that is responsible for the H57D substitution, which blocks PZA-to-POA conversion (49, 50). We generated a pH-GFP reporter M. bovis strain (Mbv pH-GFP) and assessed whether PZA-mediated pH disruption was occurring in M. bovis-infected MDM. As expected, without PZA-to-POA conversion, no drug-induced intrabacterial pH perturbation was noticeable against M. bovis (Fig. 6A and D) even at 400 mg/L. In addition, PZA intracellular activity on M. bovis replication was also investigated by quantitative fluorescence microscopy. In agreement with previous reports from *in vitro* studies (50), M. bovis was resistant to PZA *in cellulo* (Fig. 6B, C, and E). These results demonstrate that *in cellulo*, the intrabacterial pH-disruptive effect of PZA is mediated by its active form POA and that such conversion is essential for antibacterial activity within infected macrophages.

## DISCUSSION

Here, we described a novel live dual-imaging approach to monitor pH homeostasis within both the host cell and the pathogen in a biosafety level 3 laboratory. This approach is applicable to both human primary monocyte-derived macrophages (MDM) and human iPS-derived macrophages (iPSDM). In both MDM and iPSDM, ConA treatment was able to reduce M. tuberculosis-associated LysoTracker intensity, suggesting that this pharmacological inhibition is a powerful approach to perturb endolysosomal acidification in these two macrophage models. Quantitative analysis revealed that the regulation of M. tuberculosis intrabacterial pH *in cellulo* is not homogeneous with a subset of bacilli displaying a differential pH regulation as shown in Escherichia coli *in vitro* (51). At the intramacrophage population level, our data show that M. tuberculosis can maintain its own pH within endolysosomes of human macrophages, in agreement with previous findings *in vitro* and in mouse macrophages treated or not with interferon gamma (11, 31, 32). An important finding of our analysis is that impairment of endolysosomal acidification had no impact on intracellular M. tuberculosis pH homeostasis. Despite a very significant heterogeneity in LysoTracker association, most bacteria maintained their intracellular pH during the course of infection, suggesting that M. tuberculosis is very efficient at regulating intrabacterial pH in its preferred host cell.

Intracellular M. tuberculosis continuously switches between membrane-bound (able to retain protons and acidic) and cytosolic (host cell neutral-pH) localization (6), and here, by using live imaging of subcellular acidification, we tested the hypothesis that when the bacterium resides in an acidic environment, the exposure to an acidic pH will reduce intrabacterial pH. Unexpectedly, we found that irrespective of the time of residence in an acidic compartment, the bacilli maintained a constant intrabacterial pH, suggesting that mycobacteria have potent mechanisms of pH sensing and homeostasis in fluctuating environments and can rapidly respond to changes (52, 53). A limitation of our study is that we are not able to define if these bacteria in an acidic host compartment are alive and able to replicate, in a nonreplicating state, or eventually dead. More studies and technical developments are required to explore this in detail. It is also important to mention that LysoTracker staining does not allow clear discrimination between pH 4.5 and 5.5; therefore, we cannot exclude the possibility that differences might occur at these distinct pH values if bacteria reside for an extensive period of time. However, previous studies in other biological models showed that pH 5.5 or even 4.5 was not altering M. tuberculosis pH homeostasis and survival, suggesting that M. tuberculosis can adapt and survive within these conditions (11, 31, 32). We also anticipate that our system can be applied to other intracellular bacteria that temporarily inhabit acidic compartments, such as Salmonella spp., *Shigella* spp., or Coxiella burnetii.

Our dual imaging allowed us to define if, in macrophages, the localization in acidic compartments affected the intrabacterial pH after treatment with antibiotics used in the clinic to treat tuberculosis. Remarkably, out of four antibiotics, each with different modes of action, we found that in human macrophages, only PZA disrupts M. tuberculosis intrabacterial pH homeostasis in a concentration- and time-dependent manner. For BDQ, which is effective against M. tuberculosis within infected human macrophages (44, 54), we could confirm that treatment targets bacteria to acidic compartments (44), however, without resulting in significant changes in intrabacterial pH, as was previously suggested as one of the modes of action of BDQ (45) but not all the diarylquinolines (46, 47). This also suggests that intrabacterial pH imaging is not a reliable proxy of bacterial viability (at least in this system). Our findings seem to be different from what has been previously observed with Mycobacterium smegmatis, where it was proposed that global antibiotic-induced pH alterations should be considered a potential mechanism contributing to antibiotic efficacy (55).

The studies of the PZA molecular mechanism(s) of action, and its extensive association with acidic pH for efficacy, have been mostly performed *in vitro* (25, 28–30). These studies have shown that acidic environments promote PZA/POA efficacy under specific culture conditions. However, these results have also been challenged by other studies (34, 35). Indeed, inhibitory activity of PZA/POA has been reported in neutral media and in phosphate-buffered saline (PBS) buffer, suggesting that exogenous protons are not essential for the antimycobacterial action of these molecules (34, 35). Here, by using human MDM and iPSDM models, we provide compelling evidence that PZA and POA disrupt M. tuberculosis intrabacterial pH homeostasis *in cellulo*. These changes in intrabacterial pH homeostasis can be prevented by inhibiting the macrophage v-ATPase activity with ConA, likely impairing the conversion of POA^(−)^ into HPOA inside endolysosomes. The significant effect of ConA in preventing the pH disruption correlates with an increase in bacterial growth, showing that there is a link between pH disruption and antibacterial efficacy, as previously described (31). This link between intrabacterial pH alteration and efficacy seems to apply only to PZA, since the other three very potent antibiotics we tested, BDQ, RIF, and INH, are also effective against intracellular M. tuberculosis without affecting the intrabacterial pH.

Finally, by combining our dual-imaging approach with mycobacterial mutants and naturally PZA-resistant strains, we were able to define if intracellular localization affects the pH-disruption-related efficacy of PZA. Notably, two different mutants with a deficient ESX-1 secretion system showed a substantial reduction in growth compared to the wild-type strain after PZA treatment, a finding that reflects the increase in PZA accumulation reported previously (23). Indeed, nanoscale secondary-ion mass spectrometry (NanoSIMS) analysis of PZA/POA accumulation showed that two times more ΔRD1 bacteria were positive for this antibiotic in comparison to the WT strain (23), which supports the 2-fold difference in PZA EC_50_ we observed in this study. Similarly, ConA treatment negatively impacted by 3 to 4 times the amount of WT bacteria displaying a positive signal for the antibiotic by NanoSIMS (23), which was reflected by the 3.5-fold difference in antibacterial efficacy when analyzing the EC_50_ of PZA alone or in combination with ConA in the present study. Such results suggest a strong correlation between antibiotic accumulation and antibacterial efficacy as previously reported using bulk liquid chromatography-tandem mass spectrometry (LC-MS/MS) analysis (56). The use of mutants with different lifestyles in our experimental system has highlighted that cytosolic localization can be an important factor that dictates antibiotic susceptibility *in cellulo*. Our data with ESX-1-defective mutants suggest that continuous and homogenous residence in a phagosome affects sensitivity to PZA and subsequently intrabacterial pH homeostasis disruption.

Within macrophages, M. bovis was not sensitive to PZA, which did not affect intrabacterial pH, suggesting that POA, but not PZA, is primarily responsible for the pH disruption effect. This confirms that POA is the main active form of the drug and that its inhibitory effect is tightly linked to pH *in cellulo*. Importantly, our results show that PZA accumulation and efficacy require acidic environments within host cells, highlighting that the pH-dependent mechanism of action is crucial in more sophisticated biological systems (37). It is worth mentioning that the results obtained in this study are different from the one described *in vitro* by Peterson et al. (35) showing that PZA/POA did not induce pH homeostasis disruption even at pH 5.8. These discrepancies could be explained by some differing experimental parameters including differences between *in cellulo* and *in vitro* investigations, the use of H37Ra and H37Rv strains, and the increased sensitivity of fluorescence microscopy in contrast to bulk spectrofluorimetric analysis. Our quantitative imaging analysis showed that PZA-mediated M. tuberculosis intrabacterial pH perturbations *in cellulo* were significant. However, some of the effects were moderate, supporting the idea that sensitivity is an important factor to detect these changes. Our findings are in agreement with a study showing that PZA disrupts M. tuberculosis intrabacterial pH in a dose-dependent manner when incubated *in vitro* within phosphate citrate buffer pH 4.5 (31). These initial findings, obtained with an M. tuberculosis strain that expresses a pH-sensitive ratiometric GFP, were also monitored by spectrofluorimetry and confirmed (57). Finally, an alternative method relying on the use of chemical probes was able to show that POA triggers M. tuberculosis intrabacterial pH disruption in an external pH-dependent manner (32), highlighting how the results regarding PZA/POA-mediated pH homeostasis disruption remain unclear *in vitro* (36, 37).

In the last years, several studies have shown that PZA/POA can inhibit M. tuberculosis growth independently of the presence of exogenous protons by targeting the aspartate decarboxylase enzyme PanD (38–41). In this proposed mode of action, POA covalently binds to PanD and promotes its degradation by the Clp system, thus inhibiting coenzyme A synthesis (38–41). To date, little is known regarding the effect of coenzyme A depletion in M. tuberculosis with respect to cell wall integrity, global metabolism, and pH homeostasis. Therefore, more studies in our experimental system will be required to determine whether PanD inhibition or depletion could be linked to M. tuberculosis intrabacterial pH alterations. These studies have the potential to bring new insights onto PZA/POA mode(s) of action and potentially define if these compounds trigger the collapse of M. tuberculosis intrabacterial pH directly by shuttling protons through the cell wall or indirectly by impacting global homeostatic properties.

Finally, a recent study using genome-wide transposon sequencing showed that specific cellular pathways stimulated in a pH-dependent manner are linked to PZA susceptibility (58). In that context, the authors observed that the SigE-mediated response is central to conditional PZA susceptibility, adding more evidence that external pH is a critical factor that drives PZA susceptibility (58). Because M. tuberculosis displays a heterogenous subcellular localization, where a fraction of M. tuberculosis is localized in phagosomes but another fraction is in the cytosol, we postulate that dynamic and heterogenous environments contribute to the pH-disruptive action of PZA/POA in human macrophages, by potentially activating prosusceptible pathways (58) and enhancing drug enrichment within M. tuberculosis (23). The imaging approach developed here together with the concepts that have emerged from this study could be valuable to characterize antibiotic modes of action *in cellulo*.

## MATERIALS AND METHODS

### Mycobacterial strains and culture conditions.

Mycobacterium tuberculosis H37Rv and ΔRD1 strains were obtained from William R. Jacobs, Jr. (Albert Einstein College of Medicine, New York, NY, USA); Suzie Hingley-Wilson (University of Surrey, Guilford, UK); and Douglas B. Young (The Francis Crick Institute, London, UK). The Mycobacterium bovis AF2122/97 reference strain was provided by Stephen V. Gordon (University College Dublin, Dublin, Ireland). Mtb Δ*esxBA* mutant was generated in our laboratory by using the ORBIT system, genetically mapped by PCR, and sequenced (B. Aylan, E.M. Bernard, E. Pellegrino, L. Botella, C. Bussi, P. Santucci, N. Athanasiadi and M.G. Gutierrez, under review). Its respective complement Mtb Δ*esxBA*::*esxBA* was generated by transformation with a mycobacterial kanamycin-resistant integrative vector carrying a functional copy of *esxBA* genes under the control of the Psmyc promoter (Aylan et al., under review). Both clones did not show any growth impairment *in vitro* and were validated based on ESAT-6 and CFP-10 production and secretion by conventional immunoblotting (Aylan et al., under review). Recombinant M. tuberculosis or M. bovis strains expressing pH-GFP (pUV15-pHGFP; Addgene plasmid no. 70045, kindly gifted by Sabine Ehrt), red fluorescent protein (RFP) (pML2570), or E2-Crimson (pTEC19; Addgene plasmid no. 30178, kindly gifted by Lalita Ramakrishnan) fluorescent protein were generated by electroporation and further selected onto appropriate medium. Recombinant M. tuberculosis strains were grown in Middlebrook 7H9 broth supplemented with 0.2% (vol/vol) glycerol (Fisher Chemical, G/0650/17), 0.05% (vol/vol) Tween 80 (Sigma-Aldrich, P1754), and 10% (vol/vol) albumin-dextrose-catalase (ADC) (BD Biosciences, 212352) whereas recombinant M. bovis strains expressing pH-GFP or RFP were grown in Middlebrook 7H9 supplemented with 40 mM sodium pyruvate (Sigma-Aldrich, P2256). Bacterial cultures (10 mL) were incubated under constant rotation in 50-mL conical tubes at 37°C. Hygromycin B (Invitrogen, 10687010), kanamycin (Sigma-Aldrich, K1876), or zeocin (InvivoGen, ant-zn-05) was used as a selection marker for the fluorescent strains at a concentration of 50 mg/L, 25 mg/L, or 25 mg/L, respectively. All selected clones were tested for PDIM positivity by thin-layer chromatography of lipid extracts from cultures prior to performing infection experiments.

### Preparation and culture of human monocyte-derived macrophages.

Human monocyte-derived macrophages (MDM) were prepared from leukocyte cones (NC24) supplied by the NHS Blood and Transplant service as previously described (15, 23, 54). Briefly, white blood cells were isolated by centrifugation on Ficoll-Paque Premium (GE Healthcare, 17-5442-03) for 60 min at 300 × *g*. Mononuclear cells were collected and washed twice with magnetically activated cell sorting (MACS) rinsing solution (Miltenyi, 130-091-222). Cells were subsequently incubated with 10 mL red blood cell (RBC) lysing buffer (Sigma, R7757) at room temperature. After 10 min, cells were washed with rinsing buffer and then were resuspended in 80 μL MACS rinsing solution supplemented with 1% bovine serum albumin (MACS/BSA) (Miltenyi, 130-091-376) and 20 μL anti-CD14 magnetic beads (Miltenyi, 130-050-201) per approximately 10^8^ cells. After 20 min at 4°C, cells were washed in MACS/BSA solution, resuspended at a concentration of 2 × 10^8^ cells/mL in MACS/BSA, and further passed through a preequilibrated LS column (Miltenyi, 130-042-401) in the field of a QuadroMACS separator magnet (Miltenyi, 130-090-976). The LS column was washed three times with MACS/BSA solution, and then CD14-positive cells were eluted, centrifuged, and resuspended in complete RPMI 1640 with GlutaMAX and HEPES (Gibco, 72400-02), 10% fetal bovine serum (Sigma, F7524) containing 10 ng/mL of human granulocyte-macrophage colony-stimulating factor (hGM-CSF) (Miltenyi, 130-093-867). Differentiation was performed by plating approximately 10^6^ cells/mL in untreated petri dishes and further incubating them in a humidified 37°C incubator with 5% CO_2_. After 3 days, an equal volume of fresh complete medium including hGM-CSF was added. Six days after the initial isolation, differentiated macrophages were detached in 0.5 mM EDTA in ice-cold PBS using cell scrapers (Sarstedt, 83.1830), pelleted by centrifugation, and resuspended in complete RPMI 1640 medium containing 10% fetal bovine serum where cell count and viability were estimated (Bio-Rad, TC20 automated cell counter) before plating for experiments.

### Human-induced pluripotent stem cell culture and human-induced pluripotent stem cell-derived macrophage preparation.

Human iPSC maintenance and iPSDM preparation were performed as recently reported (18). Briefly, KOLF2 iPSCs (HPSI0114i-kolf_2 iPSCs; Public Health England Culture Collections, catalog no.77650100) were maintained in Vitronectin XF (StemCell Technologies, 100-0763)-coated plates with E8 medium (ThermoFisher Scientific, A1517001) in a humidified 37°C incubator with 5% CO_2_. Cells were passaged by performing a 1/6 dilution when reaching approximately 70% confluence using Versene (Gibco, 15040066). Monocyte factories were set up following a previously reported protocol (59). A single-cell suspension of iPSCs was generated in E8 medium containing 10 μM Y-27632 ROCK inhibitor (StemCell Technologies, 72307), seeded into AggreWell 800 plates (StemCell Technologies, 34815) with approximately 4 × 10^6^ cells/well, and centrifuged at 100 × *g* for 5 min. The forming embryonic bodies (EBs) were fed daily with two 50% medium changes with E8 medium supplemented with 50 ng/mL hBMP4 (Peprotech, 120-05ET), 50 ng/mL human vascular endothelial growth factor (hVEGF) (Peprotech, 100-20), and 20 ng/mL human stem cell factor (hSCF) (Peprotech, 300-07) for 3 days. On day 4, the EBs were harvested by flushing out of the well with gentle pipetting and filtered through an inverted 40-μm cell strainer. EBs were seeded at 250 to 300 per T225 flask in factory medium consisting of X-VIVO 15 (Lonza, BE02-061Q) supplemented with GlutaMAX (Gibco, 35050061), 50 μM β-mercaptoethanol (Gibco, 21985023), 100 ng/mL hM-CSF (Peprotech, 300-25). and 25 ng/mL human interleukin-3 (hIL-3) (Peprotech, 200-03). Monocyte factories were fed once per week with factory medium for 4 to 5 weeks until monocyte production was observed in the supernatant. Up to 50% of the supernatant was harvested weekly, and factories were fed with 20 to 30 mL factory medium. For differentiation, the supernatant was centrifuged and cells were resuspended in X-VIVO 15 supplemented with GlutaMAX and 20 ng/mL hGM-CSF and plated at 12 × 10^6^ cells per 15-cm petri dish to differentiate over 7 days, where a 50% medium change was performed on day 4. Seven days after the initial plating, differentiated macrophages were detached with EDTA (Gibco, 15040066) for 15 min at 37°C and 5% CO_2_. Versene was further diluted 1:3 with PBS, and cells were gently detached with cell scrapers (Sarstedt, 83.1830), pelleted by centrifugation, and resuspended in X-VIVO 15 plus GlutaMAX where cell count and viability were estimated (Bio-Rad, TC20 automated cell counter) before plating for experiments.

### Macrophage infection with M. tuberculosis and M. bovis strains.

For macrophage infection, mycobacterial inoculum was prepared following a well-established procedure (15, 42). First, bacterial cultures were pelleted by centrifuging approximately 10 mL of mid-exponential-phase cultures (optical density at 600 nm [OD_600_] = 0.6 ± 0.2) at 4,000 rpm for 5 min. Pellets were washed twice in sterile PBS buffer (pH 7.4), and then, an equivalent volume of sterile 2.5- to 3.5-mm autoclaved glass beads was added to individual pellets and bacterial clumps were disrupted by vigorous shaking. Bacteria were resuspended in the appropriate cell culture medium, and the clumps were removed by low-speed centrifugation at 1,200 rpm for 5 min. The supernatant containing the bacterial suspension of interest was transferred to a fresh tube, and OD_600_ was measured to determine bacterial concentration. In this protocol, it was assumed that an OD_600_ of 1 approximates 10^8^ bacteria/mL. For high-content dual-imaging experiments and intracellular antibiotic assays, macrophages were infected with mycobacterial strains at a multiplicity of infection (MOI) of 1 for 2 h at 37°C. After 2 h of uptake, cells were washed with PBS to remove extracellular bacteria and fresh medium was added.

### High-content dual-live fluorescence imaging, determination of M. tuberculosis intrabacterial pH-GFP fluorescence ratio, and M. tuberculosis-associated LysoTracker intensity.

For high-content live-cell imaging, cells were infected with fluorescent mycobacteria producing ratiometric pH-GFP at an MOI of 1 as described above. After 24 h, the culture medium was replaced by fresh medium only or fresh medium containing 50 nM ConA (Sigma-Aldrich, C9705) for iPSDM and 100 nM ConA for MDM. After 24 h, infected cells were washed once with PBS buffer (pH 7.4) and stained with complete medium containing 200 nM LysoTracker Red DND-99 (Invitrogen, L7528) in a humidified 37°C incubator with 5% CO_2_. After 30 min, staining medium was removed and replaced with fresh medium containing NucRed Live 647 ReadyProbes (Invitrogen, R37106) following the manufacturer’s recommendations to facilitate cell detection. Live-cell imaging was further performed using the OPERA Phenix microscope with a 63× water-immersion objective. Image acquisition was performed with the confocal mode using the default autofocus function and a binning of 1. Mtb pH-GFP signal was detected using excitation wavelength (λ_ex_) 405 nm/emission wavelength (λ_em_) 500 to 550 nm and λ_ex_ 488 nm/λ_em_ 500 to 550 nm, LysoTracker signal was detected using λ_ex_ 561 nm/λ_em_ 570 to 630 nm, and NucRed Live signal was detected using λ_ex_ 640 nm/λ_em_ 650 to 760 nm. Laser power for all channels was set between 20% and 30% with an exposure time of 200 ms. Each channel was imaged independently, and a minimum of 3 to 4 distinct focal z-planes spaced with 0.5 to 1 μm was acquired. Multiple fields of view (323 μm by 323 μm) from each individual well were imaged with a set overlap of 10% in between fields. Segmentation and analysis were performed using the Harmony software (Perkin-Elmer, version 4.9). Briefly, cellular region was detected based on the fluorescent signals in the far-red emission channel using the “Find Image Region” building block and the “Absolute Threshold” function. Intracellular Mtb pH-GFP was detected based on the GFP signal obtained into both λ_ex_ 405 nm/λ_em_ 500 to 550 nm and λ_ex_ 488 nm/λ_em_ 500 to 550 nm channels using the “Find Image Region” building block and the “Absolute Threshold” function. Signals from the two GFP channels were merged using the “Calculate Image” building block and the function “By Formula,” where a channel A+B operation was applied. This combined image was filtered to reduce background noise using the “Filter Image” building block and a sliding parabola function. This M. tuberculosis mask was used to quantify Mtb pH-GFP mean fluorescent signal per single object from both 405-nm/510-nm and 488-nm/510-nm channels. Ratiometric signals were obtained by dividing the mean intensity quantified at λ_ex_ 405 nm/λ_em_ 500 to 550 nm by the one obtained at λ_ex_ 488 nm/λ_em_ 500 to 550 nm for each object. To quantify M. tuberculosis-associated LysoTracker intensity, the M. tuberculosis mask was slightly extended using the “Find Surrounding Region” building block using method A with an individual threshold value of 0.8 and conservation of the input region. When assessing the spatiotemporal mode of action of PZA, human macrophages were infected for 24 h and further treated with increasing concentrations of PZA ranging from 0 to 400 mg/L in the absence or presence of ConA for an additional 4, 16, 24, or 72 h before acquisition was performed as described above. When assessing anti-TB drug-mediated pH disruption, M. tuberculosis-infected cells were left untreated or pulsed with PZA (100 mg/L), BDQ (2.5 mg/L), INH (5 mg/L), or RIF (5 mg/L) for 24 h before imaging. Determination of absolute changes of pH-GFP ratio relative to the control condition (also referred as Δintrabacterial pH) was done by subtracting the value obtained under each experimental condition from its corresponding control condition. All the results were exported as CSV files, imported in the R Studio software (The R Project for Statistical Computing, version 1.3.1073), and most of the graphs, displayed as boxplots, scatterplots, or raincloud plots, were plotted with the ggplot2 package (version 3.3.2).

### Low-content dual-live fluorescence imaging and live-cell imaging.

For low-content dual imaging and live-cell imaging, experimental setup and acquisition were performed as previously described (18, 42, 60) with slight modifications. Approximately 1 × 10^6^ iPSDM were seeded within 12-mm-aperture glass-bottom dishes (WillCo dish, GWST-3512) in 1 mL of X-VIVO 15 medium plus GlutaMAX. Adherent cells were infected with fluorescent M. tuberculosis producing ratiometric pH-GFP at an MOI of 1 as described above, and after 24 h of infection, medium was replaced with complete medium containing 200 nM LysoTracker Red DND-99 (Invitrogen, L7528). The dish was placed on a custom-made 35-mm dish holder and further incubated in a humidified 37°C incubator with 5% CO_2_. After 30 min of staining, the dish was set under a Leica SP5 laser scanning confocal microscope (Leica Biosystems) in an environmental control chamber providing 37°C, 5% CO_2_, and 20 to 30% humidity for an additional 1 h to avoid drifting issues upon acquisition. Image acquisition was performed with an HC PL APO CS2 63×/1.40 oil objective. Images of 1,024 by 1,024 pixels were acquired with diode 405-nm, argon 488-nm, and diode-pumped solid-state (DPSS) 561-nm lasers where intensities were set up as 2%, 8%, and 8%, respectively. Emitted signal was collected at λ_em_ 510 ± 30 nm and λ_em_ 585 ± 15 nm for pH-GFP and LysoTracker channels, respectively. One single Z-plane was acquired for each field, and a minimum of 5 fields per biological sample were imaged. Determination of the Mtb pH-GFP ratio and M. tuberculosis-associated LysoTracker mean intensity values was performed by manual quantification as previously described (18, 23). Briefly, the mROI were duplicated, the bacterium-containing channel was manually thresholded, and a single ‘Dilate’ command was applied to generate a binary mask in Fiji corresponding to the bacteria surrounded by one single ring of pixel. This mask was then used to measure the mean fluorescence intensity of pixels in λ_ex_ 405 nm/λ_em_ 510 ± 30 nm, λ_ex_ 488 nm/λ_em_ 510 ± 30 nm, and λ_ex_ 510 nm/λ_em_ 585 ± 15 nm channels using the command ‘Measure’.

For live-cell imaging a very similar experimental setup was used where images were acquired at 30-min intervals over a time frame of 6 h to minimize photobleaching and phototoxicity. The same settings were used, and 16 z-stacks of approximately 500 nm were performed to catch most of the events contained within M. tuberculosis-infected cells. mROI were defined and tracked manually by selecting the appropriate focal plane over the course of the kinetics as previously described. Selected planes were then combined using the ‘Concatenate’ command in Fiji (60). Determination of Mtb pH-GFP and its respective LysoTracker-associated mean intensity was performed as mentioned above and further analyzed over time. All the results were exported as CSV files, imported in the R Studio software (The R Project for Statistical Computing, version 1.3.1073), and the graphs were plotted with the ggplot2 package (version 3.3.2).

### Intracellular replication assays in M. tuberculosis- and M. bovis-infected macrophages.

Intracellular replication assays were performed by high-content fluorescence quantitative imaging as previously described (23, 54). Briefly, 3.5 × 10^4^ to 4.0 × 10^4^ cells per well were seeded into an olefin-bottomed 96-well plate (Perkin-Elmer, 6055302) 16 to 20 h prior to infection. Cells were infected as described above with pH-GFP or E2-Crimson-producing strains for 24 h, and the culture medium was replaced by fresh media containing increasing concentrations of PZA, RIF, INH, or BDQ or left untreated. When indicated, fresh medium containing 50 nM ConA for iPSDM and 100 nM ConA for MDM (Sigma-Aldrich, C9705) was added together with the antibiotics. At the required time points, infected cells were washed with PBS buffer (pH 7.4) and fixed with 4% methanol-free paraformaldehyde (Electron Microscopy Sciences, 15710) in PBS buffer (pH 7.4) for 16 to 20 h at 4°C. Fixative was removed, and cells were washed in PBS buffer (pH 7.4) before performing the appropriate nuclear staining using either DAPI (4′,6-diamidino-2-phenylindole; Invitrogen, D1306) or NucRed Live 647 ReadyProbes (Invitrogen, R37106) for nuclear visualization. Image acquisition was performed with the OPERA Phenix high-content microscope with a 40× water-immersion 1.1-numerical-aperture (NA) objective. The confocal mode with default autofocus and a binning of 1 was used to image multiple fields of view (323 μm by 323 μm) from each individual well with 10% overlapping, where acquisition was performed at 4 distinct focal planes spaced with 1 or 2 μm. Imaging of stained nuclei and fluorescent bacteria was done with similar λex/λem settings as described above. Analysis was performed using the Harmony software (Perkin-Elmer, version 4.9) where maximum projection of the 3 to 4 z-planes was used to perform single-cell segmentation by using the “Find nuclei” and “Find cells” building blocks. Cells on the edges were excluded from the analysis. The fluorescent bacterial signal was detected using the “Find Image Region” building block where a manual threshold was applied to accurately perform bacterial segmentation. The M. tuberculosis area per cell was determined by quantifying the total area (expressed in square micrometers) of GFP^+^ or E2-Crimson^+^ signal per single macrophage. The relative growth index was determined by using the formula (mean Mtb area per cell *t*_96h_ − mean Mtb area per cell *t*_24h_)/(mean Mtb area per cell *t*_24h_), where Mtb stands for M. tuberculosis, and the relative values were obtained by using the untreated control as a reference of 100% growth (0% inhibition). All the results were exported as CSV files, imported in the R Studio software (The R Project for Statistical Computing, version 1.3.1073), and graphs were plotted with the ggplot2 package (version 3.3.2).

### Quantification and statistical analysis.

Results displayed were obtained from *n* = 2, *n* = 3, or *n* = 4 biologically independent experiments performed at least each time in two to three technical replicates (unless otherwise stated). The statistical tests used, the number of biologically independent replicates, the number of technical replicates, and the number of single cells or single mROI analyzed are indicated in each figure legend. Statistical analysis by pairwise comparison was performed using Wilcoxon signed-rank test with the ‘*wilcox.test()*’ function in R where differences were considered statistically significant when *P *≤ 0.05. Statistical analysis is displayed in the figure as *, *P* ≤ 0.05; **, *P* ≤ 0.01; or ***, *P* ≤ 0.001, or alternatively as #, *P* ≤ 0.05; ##, *P* ≤ 0.01; ###, *P* ≤ 0.001. All the *P* values contained in the text or the figures are relative to the control condition (unless otherwise stated). Spearman rank correlation coefficient (*r_s_*) and its corresponding *P* value were calculated by using the ggpubr R package and assessed by two-tailed statistical *t* test.

### Data and code availability.

All data reported in this paper will be shared by the lead contact upon reasonable request. This paper does not report original code.
