# Supplementary material for: Visualizing Pyrazinamide Action by Live Single-Cell Imaging of Phagosome Acidification and Mycobacterium tuberculosis pH Homeostasis
Source: mBio. 2022 Mar 24;13(2):e00117-22. doi: 10.1128/mbio.00117-22 (PMC9040869; doi:10.1128/mbio.00117-22)
Supplement: FIG S1 [file mbio.00117-22-sf001.pdf]

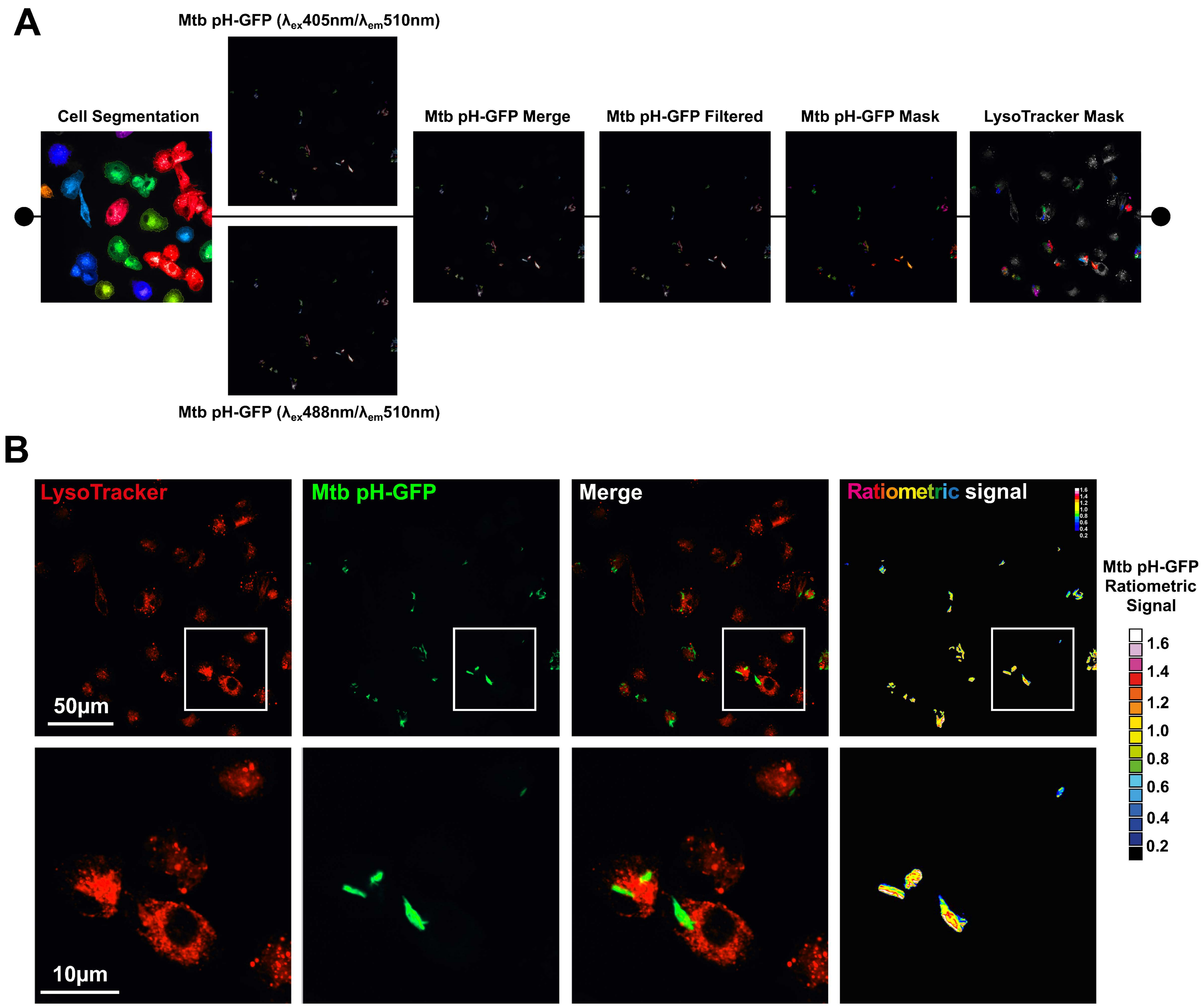

A
Mtb pH-GFP (λ_{ex}405nm/λ_{em}510nm)
Cell Segmentation
Mtb pH-GFP Merge
Mtb pH-GFP Filtered
Mtb pH-GFP Mask
LysoTracker Mask
Mtb pH-GFP (λ_{ex}488nm/λ_{em}510nm)
B
LysoTracker
Mtb pH-GFP
Merge
Ratiometric signal
50μm
10μm
Mtb pH-GFP Ratiometric Signal
1.6
1.4
1.2
1.0
0.8
0.6
0.4
0.2
