# Supplementary material for: Visualizing Pyrazinamide Action by Live Single-Cell Imaging of Phagosome Acidification and Mycobacterium tuberculosis pH Homeostasis
Source: mBio. 2022 Mar 24;13(2):e00117-22. doi: 10.1128/mbio.00117-22 (PMC9040869; doi:10.1128/mbio.00117-22)
Supplement: FIG S2 [file mbio.00117-22-sf002.pdf]

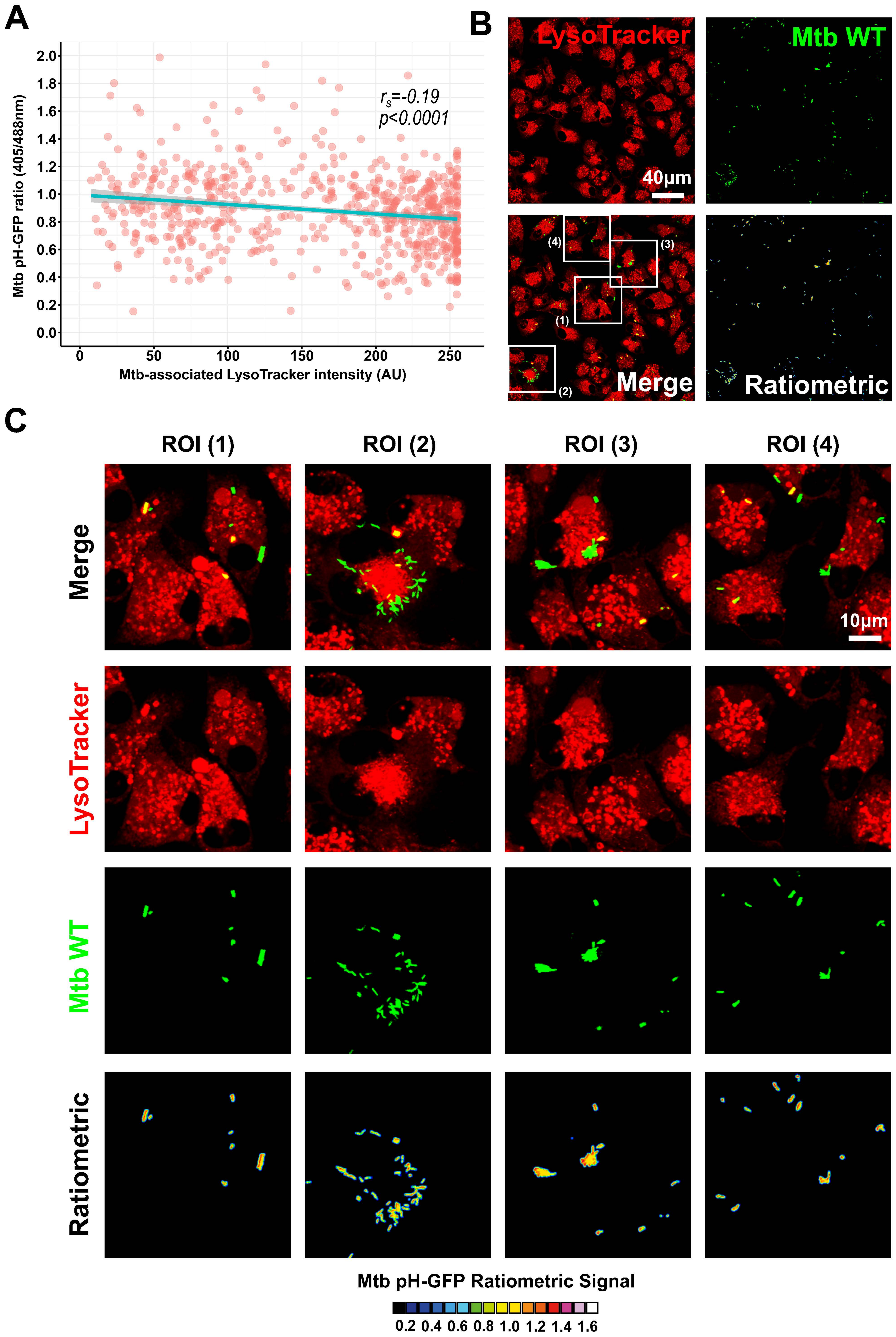
A
B
C
Mtb pH-GFP ratio (405/488nm)
2.0
1.8
1.6
1.4
1.2
1.0
0.8
0.6
0.4
0.2
0.0
0
50
100
150
200
250
Mtb-associated LysoTracker intensity (AU)
r_s=-0.19
p<0.0001
LysoTracker
Mtb WT
40µm
Merge
Ratiometric
(1)
(2)
(3)
(4)
ROI (1)
ROI (2)
ROI (3)
ROI (4)
Merge
LysoTracker
Mtb WT
Ratiometric
10µm
Mtb pH-GFP Ratiometric Signal
0.2 0.4 0.6 0.8 1.0 1.2 1.4 1.6
