# Supplementary material for: Visualizing Pyrazinamide Action by Live Single-Cell Imaging of Phagosome Acidification and Mycobacterium tuberculosis pH Homeostasis
Source: mBio. 2022 Mar 24;13(2):e00117-22. doi: 10.1128/mbio.00117-22 (PMC9040869; doi:10.1128/mbio.00117-22)
Supplement: FIG S3 [file mbio.00117-22-sf003.pdf]

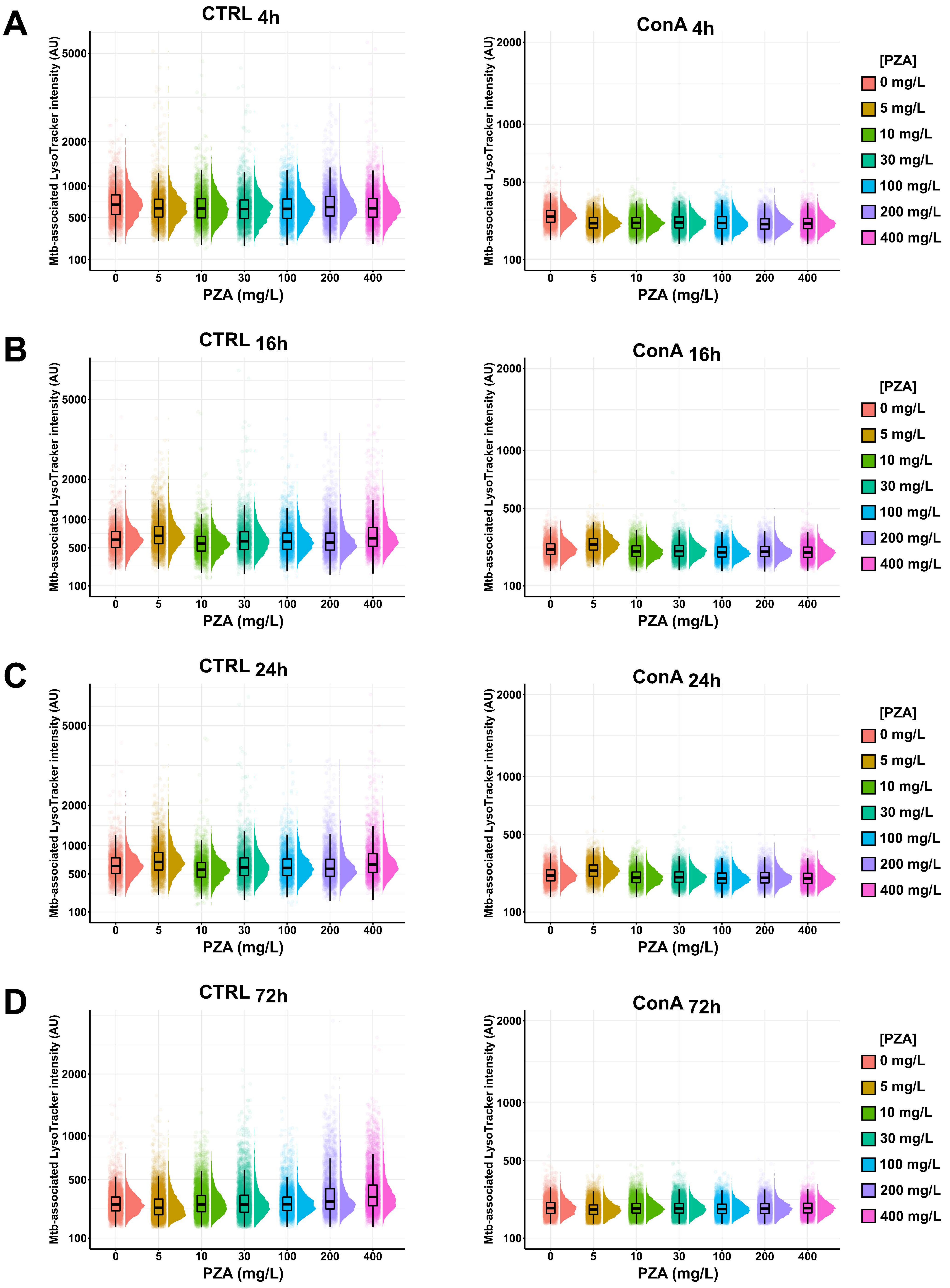
A
CTRL 4h
ConA 4h
B
CTRL 16h
ConA 16h
C
CTRL 24h
ConA 24h
D
CTRL 72h
ConA 72h
Mtb-associated LysoTracker intensity (AU)
PZA (mg/L)
0
5
10
30
100
200
400
[PZA]
0 mg/L
5 mg/L
10 mg/L
30 mg/L
100 mg/L
200 mg/L
400 mg/L
