# Supplementary material for: Visualizing Pyrazinamide Action by Live Single-Cell Imaging of Phagosome Acidification and Mycobacterium tuberculosis pH Homeostasis
Source: mBio. 2022 Mar 24;13(2):e00117-22. doi: 10.1128/mbio.00117-22 (PMC9040869; doi:10.1128/mbio.00117-22)
Supplement: FIG S4 [file mbio.00117-22-sf004.pdf]

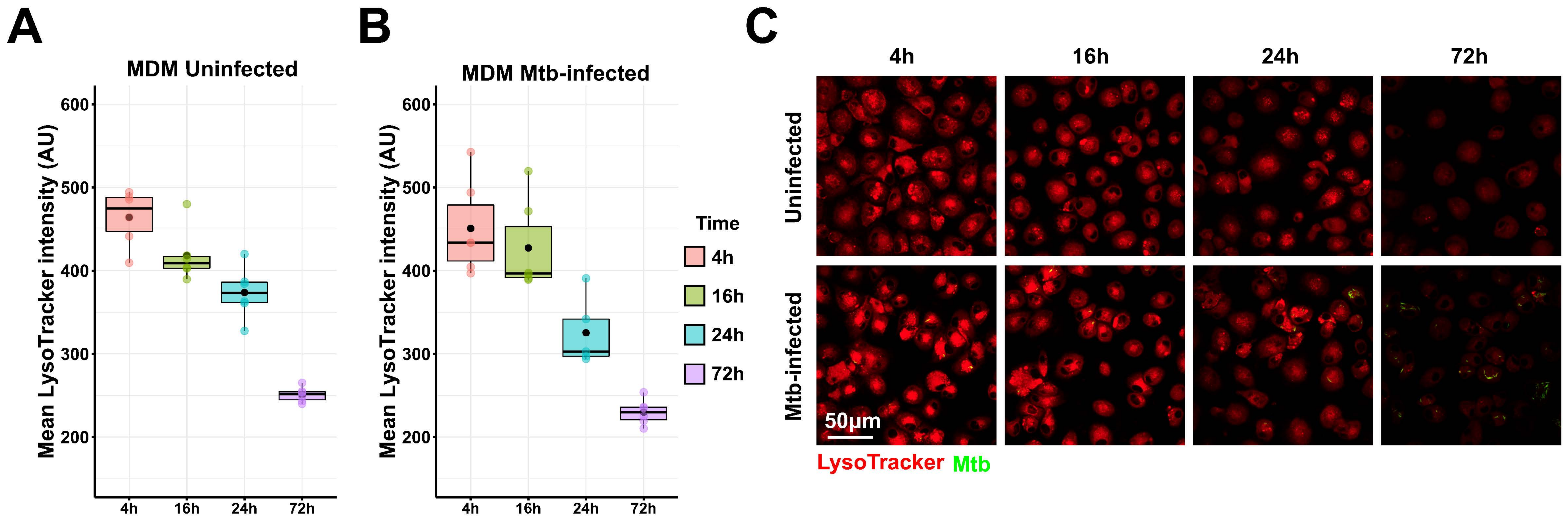

A
MDM Uninfected
Mean LysoTracker intensity (AU)
600
500
400
300
200
4h
16h
24h
72h
B
MDM Mtb-infected
Mean LysoTracker intensity (AU)
600
500
400
300
200
4h
16h
24h
72h
Time
4h
16h
24h
72h
C
4h
16h
24h
72h
Uninfected
Mtb-infected
50μm
LysoTracker
Mtb
