# Supplementary material for: Visualizing Pyrazinamide Action by Live Single-Cell Imaging of Phagosome Acidification and Mycobacterium tuberculosis pH Homeostasis
Source: mBio. 2022 Mar 24;13(2):e00117-22. doi: 10.1128/mbio.00117-22 (PMC9040869; doi:10.1128/mbio.00117-22)
Supplement: FIG S5 [file mbio.00117-22-sf005.pdf]

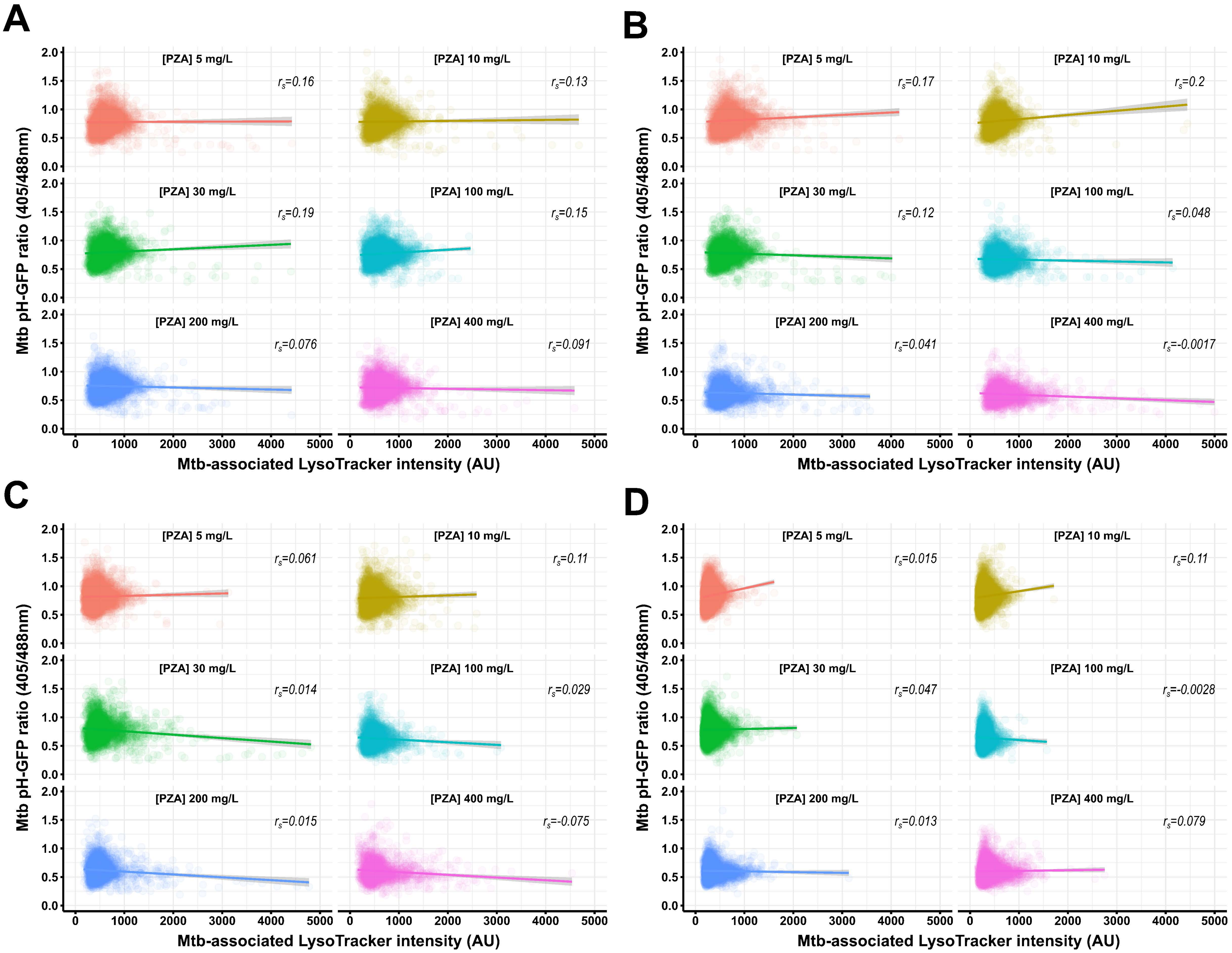

A
B
C
D
[PZA] 5 mg/L
[PZA] 10 mg/L
[PZA] 30 mg/L
[PZA] 100 mg/L
[PZA] 200 mg/L
[PZA] 400 mg/L
A: rs=0.16, rs=0.13, rs=0.19, rs=0.15, rs=0.076, rs=0.091
B: rs=0.17, rs=0.2, rs=0.12, rs=0.048, rs=0.041, rs=-0.0017
C: rs=0.061, rs=0.11, rs=0.014, rs=0.029, rs=0.015, rs=-0.075
D: rs=0.015, rs=0.11, rs=0.047, rs=-0.0028, rs=0.013, rs=0.079
Mtb pH-GFP ratio (405/488nm)
Mtb-associated LysoTracker intensity (AU)
0.0
0.5
1.0
1.5
2.0
0
1000
2000
3000
4000
5000
