# Supplementary material for: Visualizing Pyrazinamide Action by Live Single-Cell Imaging of Phagosome Acidification and Mycobacterium tuberculosis pH Homeostasis
Source: mBio. 2022 Mar 24;13(2):e00117-22. doi: 10.1128/mbio.00117-22 (PMC9040869; doi:10.1128/mbio.00117-22)
Supplement: FIG S6 [file mbio.00117-22-sf006.pdf]

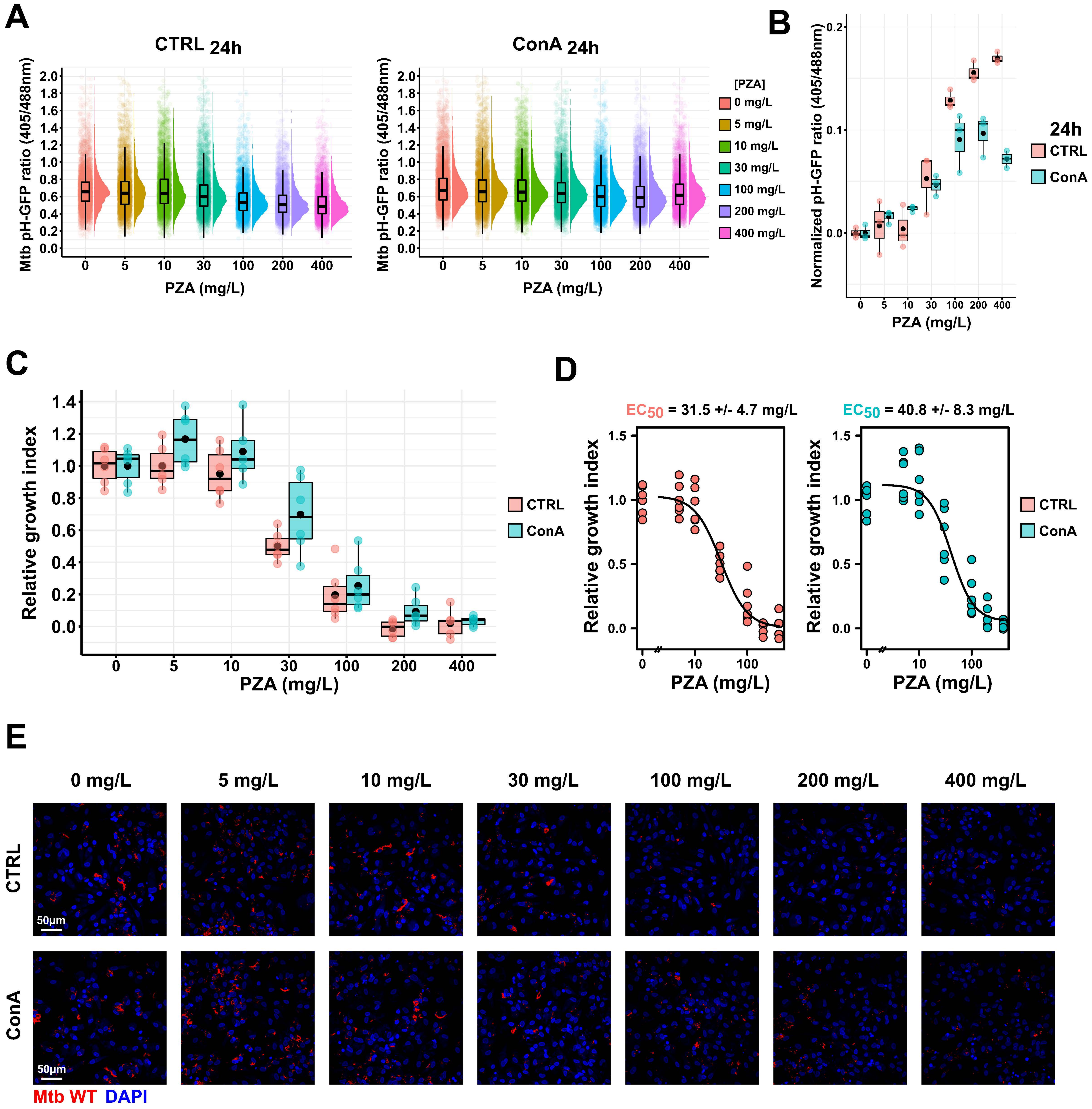
A
CTRL 24h
ConA 24h
Mtb pH-GFP ratio (405/488nm)
PZA (mg/L)
[PZA]
0 mg/L
5 mg/L
10 mg/L
30 mg/L
100 mg/L
200 mg/L
400 mg/L
B
Normalized pH-GFP ratio (405/488nm)
24h
CTRL
ConA
C
Relative growth index
D
EC50 = 31.5 +/- 4.7 mg/L
EC50 = 40.8 +/- 8.3 mg/L
E
0 mg/L
5 mg/L
10 mg/L
30 mg/L
100 mg/L
200 mg/L
400 mg/L
CTRL
ConA
50µm
Mtb WT DAPI
